# A Quantitative Re-Assessment of Microencapsulation in (Pre-Treated) Yeast

**DOI:** 10.3390/molecules29020539

**Published:** 2024-01-22

**Authors:** Giulia Coradello, Chiara Setti, Roberto Donno, Matilde Ghibaudi, Federico Catalano, Nicola Tirelli

**Affiliations:** 1 Laboratory for Polymers and Biomaterials, Fondazione Istituto Italiano di Tecnologia, 16163 Genova, Italy; giulia.coradello@iit.it (G.C.); settichiara.90@gmail.com (C.S.); mghibaudi@ibecbarcelona.eu (M.G.); 2 Department of Chemistry and Industrial Chemistry, University of Genoa, Via Dodecaneso 31, 16146 Genova, Italy; 3 Electron Microscopy Facility, Istituto Italiano di Tecnologia, Via Morego, 30, 16163 Genoa, Italy; federico.catalano@iit.it

**Keywords:** *Saccharomyces cerevisiae*, microencapsulation, terpenes, vitamin E, nutraceuticals

## Abstract

Most hydrophobes easily diffuse into yeast cells, where they experience reduced evaporation and protection from oxidation, thus allowing inherently biocompatible encapsulation processes. Despite a long-standing industrial interest, the effect of parameters such as how is yeast pre-treated (extraction with ethanol, plasmolysis with hypertonic NaCl, depletion to cell walls), the polarity of the hydrophobes and the process conditions are still not fully understood. Here, we have developed thorough analytical protocols to assess how the effects of the above on *S. cerevisiae*’s morphology, permeability, and encapsulation efficiency, using three differently polar hydrophobes (linalool, 1,6-dihydrocarvone, limonene) and three separate processes (hydrophobes as pure ‘oils’, water dispersions, or acetone solutions). The harsher the pre-treatment (depleted > plasmolyzed/extracted > untreated cells), the easier the diffusion into yeast became, and the lower both encapsulation efficiency and protection from evaporation, possibly due to denaturation/removal of lipid-associated (membrane) proteins. More hydrophobic terpenes performed worst in encapsulation as pure ‘oils’ or in water dispersion, but much less of a difference existed in acetone. This indicates the specific advantage of solvents/dispersants for ‘difficult’ compounds, which was confirmed by principal component analysis; furthering this concept, we have used combinations of hydrophobes (e.g., linalool and α-tocopherol), with one acting as solvent/enhancer for the other. Our results thus indicate advantages in using untreated yeast and—if necessary—processes based on solvents/secondary hydrophobes.

## 1. Introduction

This study provides a quantitative, comparative evaluation of some critical parameters for the encapsulation of hydrophobic compounds in yeast, whose cells act as carriers with colloidal dimensions (2–5 μm). Yeast cells can be loaded with hydrophobic compounds of nutritional, organoleptic, or pharmaceutical interest, protecting them against thermal stresses [1], irradiation [2], oxidation [3,4] or rapid evaporation [5,6]. During their loading, hydrophobes diffuse through the yeast cell wall and plasma membrane and are eventually stabilized as cytoplasmic droplets. This process does not require viable cells [5]; therefore, spent yeast from fermentation processes can also be used, despite its variable (and occasionally very low) viability, which allows for the implementation of a circular economy paradigm [7]. The hydrophobes can then be released by degrading the host cell [8], for example, through high temperature baking, which will release any encapsulated principle.

Encapsulation in yeast is an established and rather high Technology Readiness Level (TRL) process; pioneered in the 1970s [9], industrially, it has been applied predominantly to aromas and fragrances in food or textile products. Considering the translational potential of this technology, it is somewhat surprising that, in the published literature, even its most basic process parameters are hard to compare. Encapsulation efficiency is a most striking example: depending on the study, it is quantified through either the hydrophobe’s fraction remaining in the supernatants [10] or, more commonly, in the yeast pellets [5,11,12,13]. Combined, they should correspond to the original amount of material. However, this typically is not the case because (a) the former parameter (encapsulation calculated by difference) does not necessarily account for all non-encapsulated material (oil films are often forgotten), (b) the use of extraction protocols with different efficiency makes the latter parameter (directly measured encapsulation) very variable, and (c) losses from evaporation, adsorption in the container, etc., are almost never considered, and when oil films are taken into account, it is often through a rather crude determination (e.g., extraction with hexane at room temperature [13], which may also extract cytoplasmic contents). Another frequent source of confusion is that encapsulation is sometimes reported in relation to the yeast’s wet [14] or dry [12,13] weight. In short, even the mere comparison of published results is often not trivial. Therefore, it is not surprising that a rather common fragrancy such as limonene (an oily terpene from orange peel) is reported to be encapsulated in *S. cerevisiae* up to >80% wt. (against dry yeast) in Sultana et al. [15] but only 27% wt. (again, vs. dry yeast) in Errenst et al. [16], and even 5% wt. (vs. wet yeast) in Bishop et al. [14] and 3% wt. (vs. wet weight, transformed into dry by a 0.84 correction factor) in Ciamponi et al. [5].

Even more important issues are encountered when dealing with the state/morphology of yeast. Although most contributions have used specific strains of *S. cerevisiae*, this may have been (a) sourced directly from a culture [17], (b) a byproduct of industrial processes (spent, but mostly intact cells) [15], or (c) subjected to pre-treatments such as plasmolysis [18] (also known as convex plasmolysis, due to the rounder shape of the cytoplasm [19] following the cleavage of the membrane–cell wall adhesion spots [20]), treatment with organic solvents before [3] or during [21] encapsulation, or depletion of its cytoplasmic content, thus reducing it to nothing more than its cell walls [22]. There is a distinct lack of comparative studies for the different yeast pre-treatments, despite them having the potential to heavily affect the yeast barrier properties (related to the kinetics of encapsulation) and loading capacity (thermodynamics). In detail:

- Extraction with organic solvents (e.g., ethanol) partially solubilizes cell membranes [23], which should accelerate entrance, but—by removing intracellular lipids—it may also reduce encapsulation capacity.
- Plasmolysis via hypertonic shock produces invaginations on the plasma membrane [24] and increases the distance of the latter from the cell wall, separating the two irreversibly [19]. It may remove some cytoplasmic content [25], but is unlikely to seriously affect intracellular lipid content, and therefore hydrophobe encapsulation may not be seriously affected (lipid content is its thermodynamic driving force [26,27]). Plasmolysis may therefore affect the kinetics rather than the overall efficiency of the process; actually, there are reports that efficiency was actually increased by plasmolysis [11,13], as well as others claiming that it was not [3,12].
- Depletion leaves only one barrier in place (cell walls) and removes intracellular lipids (via saponification) [7], thus it seems reasonable to expect an even more accelerated permeation and a very reduced loading.

With all this in mind, it is remarkable that only a few quantitative studies have been conducted to compare the performance of differently treated yeasts [23,28], and—to our knowledge—clear links between treatments and their effects on yeast structure and on encapsulation have hardly been established.

**Scheme 1:**
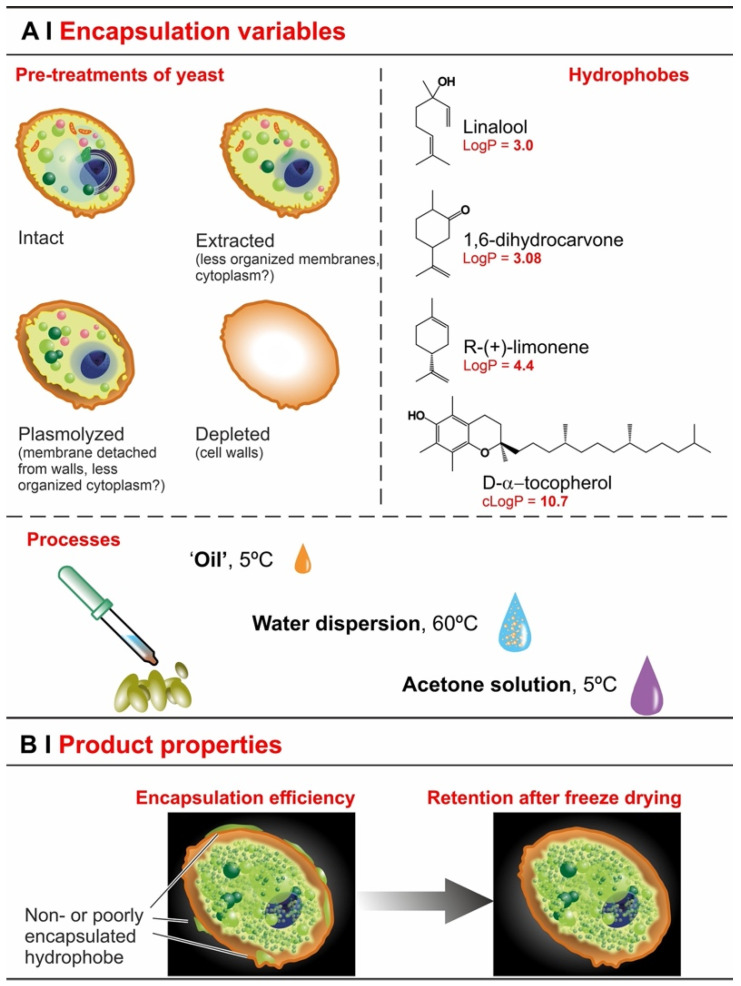
In this study, we have screened three variables (how yeast was pre-treated, the nature/hydrophobicity of the compound to encapsulate, and the process employed) in terms of their effects on two properties of the loaded yeast (how efficiently the hydrophobes were encapsulated, how firmly they were retained upon lyophilization). Please note that the volatility of hydrophobes is supposed to be markedly different depending on whether they will be located within the (significantly restructured) cytoplasm, or in their periplasmic space/outside the cell wall.

Here, we have developed a rigorous analytical procedure to explore a ternary variable space, which bundles together three orthogonal parameters (Scheme 1): the pre-treatment (intact vs. plasmolyzed, extracted or depleted cells, and what this means for the structure and permeability of the yeast cells), the kind of process (based on pure hydrophobe (‘oil’), water dispersion, organic solution), and the hydrophobicity of the ‘active’ compound (three monoterpenes, i.e., (R)-(+)-limonene, linalool and 1,6-dihydrocarvone, and one terpenoid, i.e., α-tocopherol). The main aim of this study was to link the above variables to the overall encapsulation efficiency and to the retention of hydrophobes under vacuum; the latter is as a countercheck for the ‘real’, functional encapsulation, as opposed to, e.g., surface adsorption, which would allow for easier evaporation.

Further, we have explored the possibility of using mixtures of hydrophobes, specifically focusing on the combination of a ‘difficult’ compound (α-tocopherol, viscous and very hydrophobic, and hence not amenable to use in water dispersions or as a pure ‘oil’) with an ‘easy’ one (linalool; low viscosity, amenability to all processes, high encapsulation efficiency). In a way, this may be seen as a form of solution encapsulation, where one can obtain the synergy of the latter compound acting as a carrier for the former. However, antagonism can also occur, i.e., the former blocking the latter or the two competing for the same intracellular pockets. It is also worth mentioning that, from the perspective of using yeasts of different origins (e.g., spent, or from various commercially available sources), we have also looked at the effects of washing and drying steps, in order to ensure that these ‘conditioning’ phases do not introduce further sources of variability.

## 2. Results

### 2.1. Yeast Conditioning

**Figure 1:**
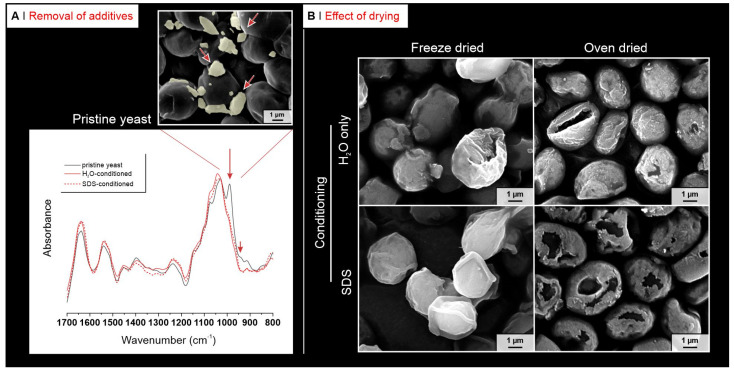
(**A**) Commercially available yeast often contains stabilizers that appear as small debris in SEM pictures (colored in light yellow and highlighted with red arrows in the inset). Two IR peaks (below 1000 cm^−1^, which may be tentatively assigned to C-O stretching vibrations, highlighted by red arrows) disappear upon washing; please note that the peaks do not coincide with those of yeast saccharides above 1000 cm^−1^. (**B**) SEM analysis showed no aggregates or debris after washing (with or without SDS); yeast cells were significantly damaged by oven-drying but not by freeze-drying.

Spent or commercially available yeast is often stabilized with excipients such as preservatives or cryoprotectants, whose aggregates can be visible in SEM pictures (inset in Figure 1A). We have first tackled the issue of ‘conditioning’, which is performed in order to make the yeast into an easy-to-handle dry powder made of yet-intact cells. Repeated washings with deionized water or with a surfactant (SDS) solution removed the aggregates and caused an IR band at 989 cm^−1^ to disappear (Figure 1A), which suggests the excipients to be polyols or polyethers used as cryoprotectants (supplier documentation confirms: sorbitan monostearate). Of note, the way the yeast was dried, as a second phase of this conditioning process, was critical for the integrity of the yeast cells (Figure 1B): drying in an oven at 0.01 mbar/40 °C/24 h caused extensive cracking, whereas freeze-drying left the cells slightly shrunken but intact. The washing and drying conditions did not differ significantly in terms of their effects on yeast ultrastructure and, specifically, the distribution of intracellular lipid bodies (see Supporting Information, Figure S2).

### 2.2. Yeast Pre-Treatment

Conditioned yeast was subjected to pre-treatments, which affected both barriers (membrane, cell wall) and hydrophobe-hosting structures (intracellular organelles, lipid droplets, and membranes) in an increasingly aggressive fashion: plasmolysis using a hypertonic solution (20% NaCl/45 °C/2 h) caused membrane poration and osmotic shock; extraction with ethanol (50% EtOH/room temperature/2 h) reduced the cell membrane barrier function and significantly degraded cytoplasmic structures; and depletion via saponification (1M NaOH/85 °C/1 h, Ph = 4.5/60 °C/1 h, then isopropanol) left behind only the cell walls.

**Figure 2:**
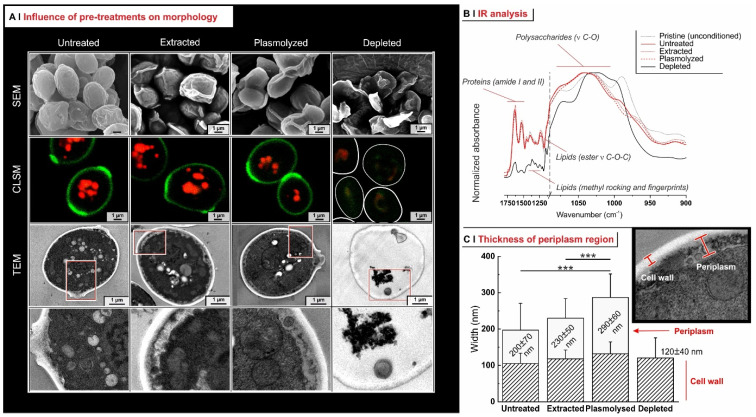
(**A**) SEM, confocal microscopy (red: lipid bodies stained with Nile Red; green: cell wall mannoproteins stained with concanavalin A-Alexa Fluo 488), and Brightfield-TEM pictures (with magnifications of the contoured areas) of yeast before and after the three pre-treatments. Although confocal images suggest that extraction and plasmolysis do not introduce significant changes, SEM and TEM, respectively, showed increased external wrinkles and extended the periplasmic space. Depleted cells, on the other hand, appear almost completely devoid of cytoplasmic content, and a faint concanavalin stain on their walls (white perimeter from brightfield images). (**B**) Infrared spectra normalized at 1040 cm^−1^ (peak of alcohol/ether of polysaccharide C-O stretching vibration (υ_C-O_)) show a sharp reduction in both proteins (amides) and lipids in depleted yeast, and also a different structure of the υ_C-O_ band. (**C**) The thickness of cell walls and of the underlying periplasmic spaces (see inset) can be estimated from TEM images; a 2-way ANOVA analysis shows significant differences between the average thickness of the periplasmic space in plasmolyzed cells and those of untreated and ethanol-extracted cells (*n* = 40 with 8 measuring points per cell; *p* = 0.005 (three stars)). Additional images are provided in the Supporting Information, Figure S4.

Plasmolysis and ethanol extraction did not appreciably modify yeast morphology and composition (Figure 2A): external appearance (SEM pictures) and cytoplasmic organization (TEM pictures) were not significantly affected, nor was the number of lipid droplets per cell (CLSM pictures), which tallies with a comparable Nile Red emission intensity (see Supporting Information, Figure S5A). However, the two treatments did have measurable effects: membranes were damaged by ethanol extraction and, slightly less, by plasmolysis, e.g., they showed a monolayer structure in certain areas (Figure S4). The periplasmic thickness (wall–membrane distance; Figure 2C) also increased, especially in plasmolyzed cells. Unsurprisingly, depletion had more dramatic effects: cytoplasm was essentially removed (TEM), and lipids and mannoproteins disappeared (CLSM stains) or were strongly reduced (Figure 2B: IR peaks associated to amide I/II of proteins and methyl rocking/C-O-C stretching of lipids are almost absent, which is different from C-O stretching of cell wall polysaccharides). Furthermore, depletion markedly increased water uptake, decreasing mass density (higher number of cells per weight unit) and opacity due to the absence of scatterers, such as organelles and membranes (see Supporting Information, Figure S3). In short, depletion essentially converted the yeast to something hardly more than its own cell walls, though it did not alter the thickness of the latter (Figure 2C).

### 2.3. Effects of Pre-Treatments on Yeast Permeability

**Figure 3:**
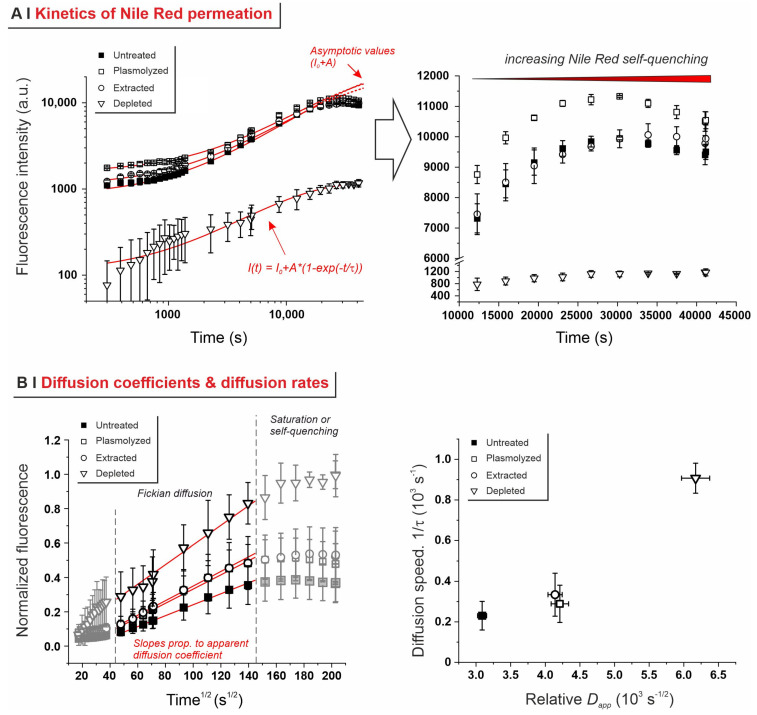
(**A**) Nile Red fluorescence intensity (ex. 545 nm/em. 620 nm) plotted as a function of time for differently pre-treated yeasts (**left**); Nile Red emission is quenched in the surrounding water medium and the signal is only attributable to fluorophore that has penetrated into the cells. The Nile Red emission can be fitted as a function of time with a single exponential model (equation and curves in red; see Experimental Section for further details); the model closely fits the experimental data of depleted yeast but deviates from those of the other pre-treated yeasts after 45–50 min, when emission starts to decrease (**right**). This could be attributable to Nile Red intracellular self-quenching. For these yeast samples, experimental data were fitted only up to 45 min, and the asymptotic emission values were then extrapolated (dashed red lines in the left graph). *n* = 3. (**B**). In order to estimate the apparent diffusion coefficients, the fluorescence intensity at 620 nm was normalized against the asymptotic emission, i.e., the sum I_0_+A as obtained through the single exponential fitting, and reported against the square root of time (**left**). The initial and the last parts of the resulting graphs (grey symbols) were not used for calculations because, respectively, the data were too noisy, or they deviated from linearity due to saturation (for depleted yeast) or self-quenching effects (for all other yeasts). The slopes of the central parts of the graphs (in red) are directly proportional to the Nile Red apparent diffusion coefficients D_app_. A correlative graph (**right**) between Nile Red diffusion speed (the reciprocal of the fitted parameter τ refers to the whole process) and its apparent diffusion coefficient (expressed in a relative form as the slopes of the central part of the process) highlights the progressive permeabilization of the yeast cells.

These effects were assessed using the lipophilic fluorophore Nile Red. Initially used for mammalian cells [29], Nile Red is now popular to quantify lipid bodies in yeast [30], being amenable to high-throughput methods and less cumbersome to classical lipid analysis via cell lysis/extraction [31]. Importantly, it is quenched in water, therefore the time dependency of its emission is a direct measure of its diffusion into intracellular hydrophobic pockets. This kinetics followed an exponential behavior for up to 5–6 h (Figure 3A, left), whereas at longer exposure times, this fitting was appropriate only for depleted yeast; all other yeast types deviated, probably due to Nile Red self-quenching (Figure 3A, right). By limiting the results to those obtained during the first time period, one obtains a characteristic permeation time τ, whose reciprocal is effectively a diffusion speed.

As a second numerical indicator, an apparent diffusion coefficient D_app_ can be obtained from the region of Fickian diffusion, i.e., the time period in which internalization proceeds linearly with the square root of time (central part of the kinetics in Figure 3B, left). In this area, the slope of the graph is proportional to a D_app_ through morphological parameters (the actual shape and dimension of the yeast cells) and the number, integrity, and thickness of the barriers (walls and membranes, if any). Please note that D_app_ is not a well-defined diffusion coefficient, due to the heterogeneity of the barriers to overcome. The diffusion speed 1/τ and the relative D_app_ both indicated that depletion caused massive permeabilization (Figure 3B, right); D_app_ also highlighted that plasmolyzed and extracted cells were more permeable than intact ones, a difference not appreciable in the noisier 1/τ data.

### 2.4. Encapsulation Processes for Single Hydrophobes

**Figure 4:**
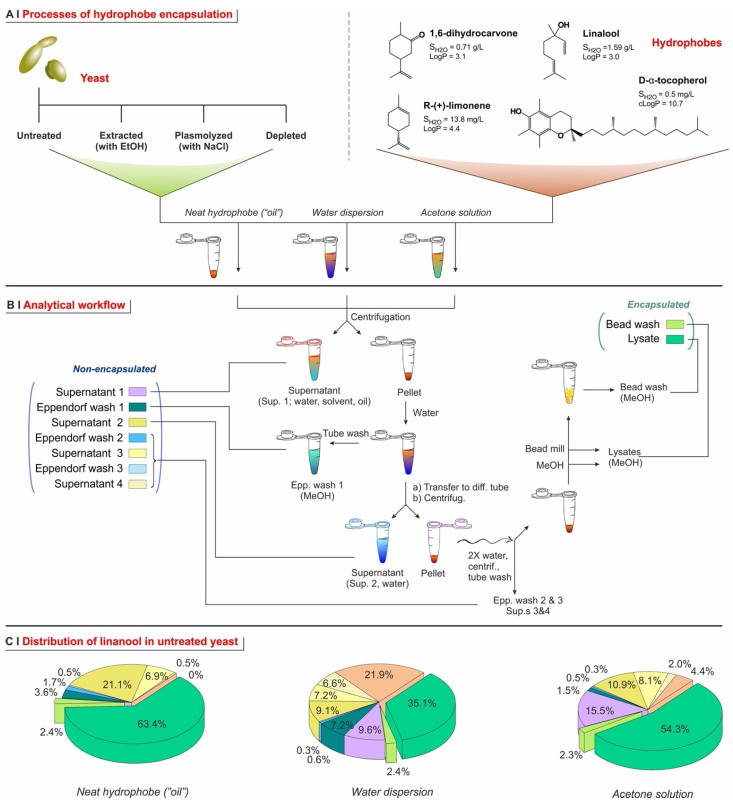
(**A**) Three terpenes (1,6-dihydrocarvone, linalool, limonene) and one terpenoid (α-tocopherol, always used in mixture with linalool or acetone in order to reduce its viscosity) were encapsulated in four yeasts (untreated/control, plasmolyzed, extracted, and depleted yeast cells) using three different methods (encapsulation with neat hydrophobe, water dispersion, or acetone solution). The logP values of 1,6-dihydrocarvone, linalool, and limonene are taken from literature sources, while that of α-tocopherol was calculated; S_H2O_ values are the solubilities of these compounds in water at 25 °C. (**B**) For all processes, we separately assessed the amount of encapsulated and non-encapsulated material. For the former, yeast cells were destroyed in a bead mill using glass beads, and the hydrophobes were extracted separately from the cell lysates and from the beads with methanol. For the quantification of the non-encapsulated material, the workflow employed several steps of centrifugation and washing (with water) of the encapsulated yeast, and of extraction (with methanol) of the plasticware. (**C**) The analytical workflow described in *B* assessed the amount of payload present in nine fractions (two for encapsulated material, the others accounting for different fractions of the non-encapsulated hydrophobe). The difference between the initial amount of hydrophobe used and their recoverable quantity (the nine fractions combined) is ascribed to loss due to evaporation (in the pie charts, this is color-coded in orange); this discrepancy is almost negligible for low-temperature processes (organic solution, ‘oil’), whereas about 22% of the hydrophobes were lost during encapsulation in water dispersion (carried out at 60 °C). Please also note that the % amounts are rounded to the lower decimal figures, therefore the overall sum is not perfectly 100%. For each fraction, *n* = 3.

In this comparative evaluation, we have paid specific attention to the analytical workflow (Figure 4A) for an absolute quantification of the recoverable payload vs., e.g., evaporative losses. For each experiment, we have assessed a total of nine different fractions (color-coded in Figure 4B), thereby taking into account any form of encapsulated (within yeast cells) and non-encapsulated (supernatants/adsorbed on plastic tubes/in yeast washings) material and allowing for a reliable comparative assessment of the performance of the various encapsulation conditions (Figure 4C). It is noteworthy that more than 20% of the hydrophobe may evaporate (e.g., when processed at 60 °C, as in the water-based process described later), and 5–15% of it was found adsorbed on plastic or glassware (e.g., Eppendorf tubes, beads used to lyse yeast cells).

In this study, we have employed three liquid hydrophobes with similar molecular weights but different structures and polarities: the logP of linalool is similar to that of 1,6-dihydrocarvone (both around 3), but the structure of the two molecules is different (the former is linear, the latter cyclic); conversely, limonene is cyclic as 1,6-dihydrocarvone, but considerably less polar (logP: 3.8). These three compounds were tested on untreated and pre-treated yeast, screening the efficacy of the following encapsulation procedures: directly as pure liquids (‘oil’), dispersed in water, or dissolved in a membrane-permeable and easy-to-remove solvent (acetone).

Other parameters were also taken into account in this screening phase:

- Temperature. The encapsulation from water dispersion was performed at 60 °C. Such relatively high temperatures are routinely used in these processes [14,23] because the lower viscosity and higher diffusion coefficients of both water and hydrophobes, as well as the higher membrane fluidity [32], are believed to allow for a better/more rapid encapsulation; losses due to hydrophobe evaporation are sizeable but rather limited (up to around 10–15% in weight, see Figure 4C). On the other hand, when in pure ‘oil’ or acetone, a high temperature would entail excessive evaporation, thus these processes were operated at 5 °C, a temperature also used elsewhere for similar encapsulation protocols [7].
- Identity of the solvent (only for acetone encapsulation). The use of an organic solvent has two potential advantages: it may increase the solubility of the hydrophobes in the yeast cell walls (as we have demonstrated for the non-volatile DMSO [5], which requires a more cumbersome removal), but it also reduces the viscosity and increases the volume of the hydrophobic phase, which allows for more homogeneous mixing with the yeast pellets. In the literature, ethanol is often used for this purpose, either pure [12] or in a 50% water mixture [23]. Here we have shown that, in linalool-based pilot experiments, acetone alone determines a higher encapsulation (see Supporting Information, Figure S6) and was thus used in all the encapsulation experiments from an organic solution. It is noteworthy that, as encapsulation from acetone was significantly higher than from ethanol, and the latter only slightly better than water slurry, this order qualitatively agrees with the dielectric constant of the carriers (66.7 [33], 29.1 [34], <19.45 [35], respectively, for water, ethanol, and acetone).
- *Hydration of the yeast pellet.* Cell wall hydration has been reported to be essential for permeation through yeast cell barriers (cell wall and plasma membrane) [15,26]. Using acetone solutions (5 °C), linalool encapsulation was indeed somehow more efficient on pre-wetted yeast (see Supporting Information, Figure S6). When using water dispersion (60 °C), there was no significant difference between dry and wet yeast, which is not surprising because its cells would be hydrated in situ by the dispersing medium. Using a neat hydrophobe (‘oil’), encapsulation efficiency was high for both wet and dry yeast, marginally better for the latter, possibly because of the more difficult mixing of an oil with a wet ‘cake’ of yeast. Therefore, further water dispersion and acetone solution experiments were performed on hydrated yeast, while those using oil were performed on dry yeast.
- *Amount of solvent (encapsulation from ethanol or acetone).* We have used linalool with identical doses (15 mg per 50 mg of yeast) but different concentrations, i.e., varying only the volume of solvent (acetone or ethanol; either pure or 50% in water; see Supporting Information, Figure S6). With pure solvents (no water), encapsulation efficiency increased proportionally to linalool concentration, which suggests a simple partition. Conversely, for 50% aqueous solvents, efficiency was generally higher at lower linalool concentrations, which hints to the solubility of the hydrophobe in the cell walls—remaining low because of their hydration—being the controlling factor. For further experiments, we have chosen to be under partition (thermodynamic) control, and therefore employed acetone (performing better than ethanol) as a pure solvent. Of note, we have, however, employed the second (120 mg/mL, corresponding to 2.5 µL solution/mg yeast) and not the first highest concentration, due to its better ability to wet the yeast pellets.
- *Repeated additions.* It has been reported [7] that repeated encapsulation cycles on the same sample may increase the overall loading capacity. We have, however, observed the opposite. A single exposure at a dose of 15 mg of linalool per 50 mg of yeast, at different concentrations in pure acetone, had a much higher encapsulation efficiency than five successive cycles under the same conditions (see Supporting Information, Figure S6). Interestingly, the latter corresponded to an overall combined dose of 75 mg of linalool per 50 mg of yeast, yet they resulted in a lower loading capacity, i.e., a lower content of linalool per yeast cell. This might be due to the extraction of a previously retained hydrophobe every time the pellet is exposed to fresh organic solvent, probably favored by the increasingly damaged cellular barriers. Therefore, for further experiments, we have only used single-addition experiments.

**Figure 5:**
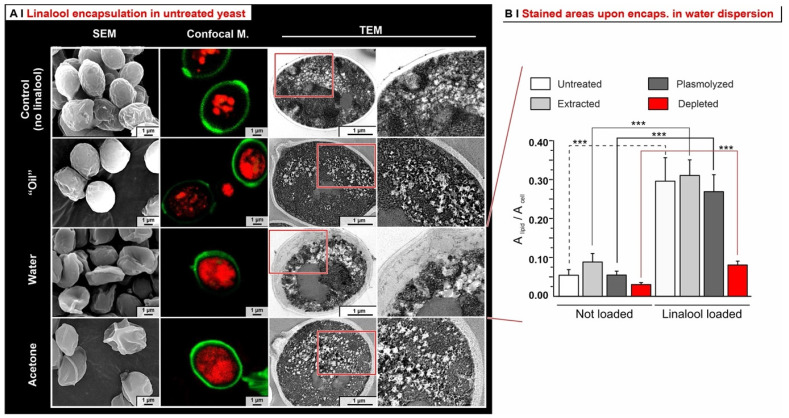
(**A**) Yeast external and internal appearance, as probed via SEM (first column from the left), confocal microscopy with lipid stain (second column from the left), and TEM (two columns on the right) after linalool encapsulation in untreated cells. Of note, after encapsulation at 60 °C from water dispersion, the distance between cytoplasmic elements and cell membrane on one side and cell walls on the other (the periplasmic space) is significantly larger, which may be ascribed to the higher temperature of the treatment. (**B**) Upon encapsulation of hydrophobes (in this case linalool, encapsulated from water dispersion), the Nile Red-stained areas occupy a larger proportion of the cell body. This Nile Red- stained fraction of the cell body increases markedly (3–5 times) for untreated, extracted, and plasmolyzed yeast, but to a lower extent (about 2 times) for depleted yeast; this tallies with the lower encapsulation efficiency of the depleted cells (see Figure 6). One-Way ANOVA with Bonferroni correction, *p* = 0.001 (three stars), *n* > 13.

Following the above experimental considerations, we have assessed the effects of the three encapsulation processes on yeast cell morphology, using linalool as the model compound (Figure 5A). No process appeared to significantly damage cell surfaces (SEM), or alter cell dimensions (both SEM and confocal microscopy, see Supporting Information, Figure S7), but Nile Red-stained lipid areas considerably increased (see Figure 5A for pictures of untreated yeast, and Figure 5B for data of all pre-treatments). It is noteworthy that a higher loading corresponded to intracellular fluorescence losing a punctuated morphology and becoming diffuse (Supporting Information, Figures S8 and S9), suggesting the disruption of well-defined lipid bodies. Finally, TEM pictures showed encapsulation to cause a ‘granulation’ of the cytoplasmic space, with enlarged and more numerous lipid bodies and a difficult identification of any other organelle. In terms of specific encapsulation processes, we noted that, when using water dispersions, the distance between the cell wall and cytoplasmic components significantly increased; this may be an effect of the combination between the presence of the hydrophobe and the higher temperature of the process, leading to complete membrane disruption and cytoplasm compaction. Furthermore, the acetone process may lead to a finer cytoplasmic ‘granulation’.

**Figure 6:**
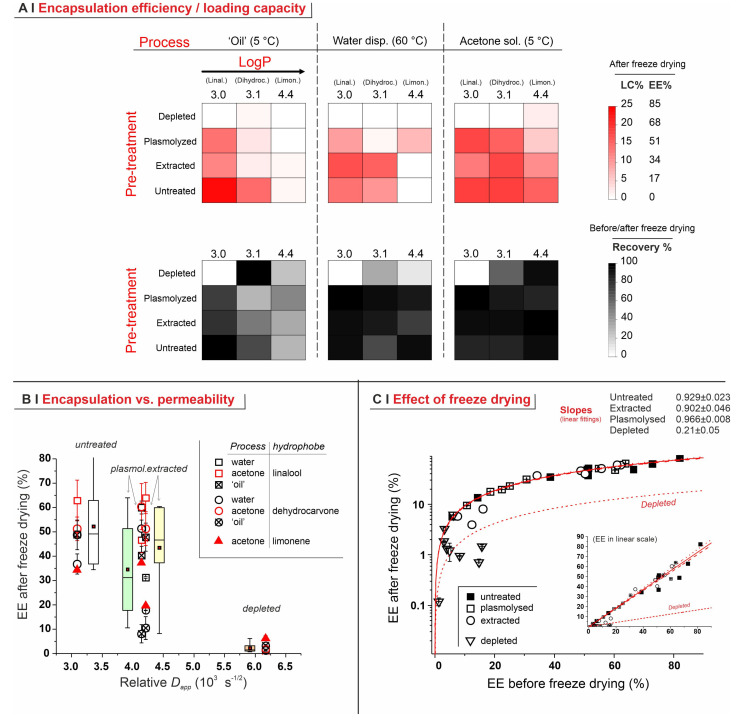
(**A**) LC% and EE% (above; from cell lysates after freeze-drying) and percentage of recovered hydrophobe (below; =ratio of hydrophobe contents before/after freeze-drying) for linalool, 1,6-dihydrocarvone, and limonene in the four differently treated yeast types and the three processes. Numerical data are available in the Supporting Information, Tables S5 and S6. Please note that the hydrophobe/yeast weight ratio is constant (here: 0.3), hence hydrophobe encapsulation efficiency (EE%) and yeast loading capacity (LC%) are necessarily proportional (LC% = 0.3 × EE%), and thus also the single color-coding with two scales. (**B**) EE% (after freeze-drying) is correlated to the Nile Red apparent diffusion coefficients D_app_ (Figure 3B), which is used as a measure of yeast cell permeability to hydrophobes. Please note that (a) the data are presented as symbols, but also aggregated as box plots (square symbols: median; central line: mean; white: untreated, green: plasmolyzed, yellow: extracted)) to better appreciate where the data of differently pre-treated yeasts are spread; (b) limonene encapsulation from water or ‘oil’ is not included because it is extremely low in all settings. (**C**). The EE% after freeze-drying is reported in a semi-log plot (linear plot in the inset) for all hydrophobes and processes, as a function of the EE% measured before freeze-drying; please note that the ratio of the two EE% is also reported in the panels at the bottom of panel A (‘recovery’). All systems, bar depleted cells, fit well with linear models having slopes around 0.9, which corresponds to losing around 10% of the encapsulated material upon evaporation.

We then proceeded to a quantitative side-by-side comparison of the amount of hydrophobes remaining after freeze-drying (in the four differently treated yeasts, using the three different processes; top panels in Figure 6A) in order to focus on ‘real’ intracellular encapsulation without considering hydrophobes adsorbed on cells or entrapped between them:

1. *Depleted cells*: they are largely incapable of hosting any of the hydrophobes (see also Figure 5B), probably because of the depauperation of lipid components, as shown by Nile Red fluorescence.
2. *Untreated cells*: with a pure hydrophobe phase (‘oil’ or water dispersion), the encapsulation efficiency decreased in the order linalool (loading capacity up to 25% wt.) > 1,6-dihydrocarvone > limonene; the markedly better performance of linalool vs. 1,6-dihydrocarvone suggests that the linear/cyclic structural difference is possibly more important than the overall polarity, which would be an important correction to what was reported by Dardelle et al. [36] (i.e., logP being the key property). The trend was less apparent when the three hydrophobes were dissolved in acetone; it is indeed reasonable that a carrier capable of increasing solubility in cell walls and helping permeation through membranes would homogenize the performance of hydrophobes differing in structure and solubility.
3. *Plasmolyzed and extracted cells*: in these two kinds of pre-treated yeast, generally linalool encapsulated best and limonene worst when using a pure hydrophobic phase, whereas no significant difference was recorded with acetone solutions. This is qualitatively similar to untreated cells, albeit mostly with a lower efficiency.

Another technologically important piece of information is the mass loss due to evaporation during freeze-drying; the three hydrophobes used in this study had a limited volatility (boiling temperatures: linalool 198 °C, 1,6-dihydrocarvone 222 °C, limonene 176 °C), yet sufficient to differentiate encapsulated from non-encapsulated material. For all cells but the depleted ones, losses upon freeze-drying were always below 10% (Figure 6A, bottom panels); independently of the encapsulation process and of the identity of the hydrophobe, the EE% before and after freeze-drying were always very similar (slopes just below 1 in the semi-log graph of Figure 6C). On the contrary, up to 90% of the loaded material evaporated from depleted cells, which suggests a direct link between lipid levels and evaporation.

These conclusions can be confirmed using a Principal Component Analysis (PCA) approach. PCA was carried out by expressing the categorical variables (i.e., the key parameters identifying the experiments) through the following numerical attributes: the individual hydrophobe via its LogP values, the pre-treatment via the Nile Red D_app_ (i.e., with their effects on wall/membrane permeability), and the encapsulation processes via the dielectric constant of the medium (see Supporting Information, Table S7). Numerical values were then also used for the main responses, i.e., encapsulation efficiency after drying and recovery of the hydrophobe, therefore providing five descriptors that identify each encapsulation experiment. Developing a set of five orthogonal Principal Components (PCs), i.e., linear combinations of the variance of all five aforementioned descriptors, it is possible to see that the first two components (PC1 and PC2) already explain 67% of the total variance, although the process of encapsulation is not well represented (only 30%). Through the addition of PC3, the explained variance reaches 87% and takes encapsulation fully into account (now explained at 95%).

**Figure 7:**
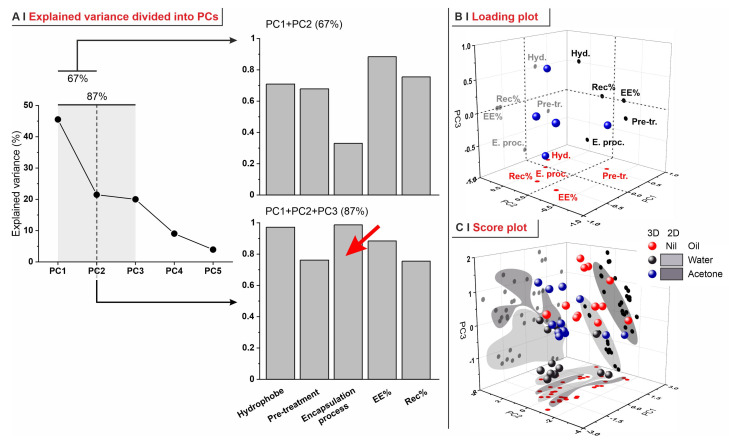
(**A**) PCA conducted on the experiments described in Figure 6A. Although the first two PCs explained 67% of the variance (**left**), the encapsulation process (represented by the dielectric constant ε of the medium) was not well represented (**top**), whose variance was, on the contrary, well explained through the first three PCs ((**bottom**), red arrow indicating the enhanced explanation of ε). (**B**) Loadings were plotted against the three main PCs, highlighting their projections on each plane. PC1 vs. PC2 (red projections on the horizontal plane) explains most of the experimental variance with a limited contribute of the encapsulation process. PC1 vs. PC3 (grey projections on the left vertical plane) instead explains a similar amount of variance, but it arises from the process and hydrophobe. PC2 vs. PC3 (black projections on the right vertical plane) is dominated by the encapsulation process. (**C**) The 36 experiments can be tridimensionally represented in the PC1/PC2/PC3 space in a score plot, color-coding them according to the encapsulation process and using spheres for the tridimensional space and dots/areas for the projections on planes. In all cases three populations can be clearly identified.

Loading plots define the positions of each descriptor in the PC space; when descriptors correlate, they are spatially close, inverse correlation implies symmetry in respect to the origin, no correlation a 90° angle. In our loading plot (Figure 7B), it is possible to see the following:

(A) In PC1 vs. PC2, recovery was inversely correlated to pre-treatments and not correlated to the hydrophobe identity; this reflects the lower retention capacity of the most permeabilized yeasts (depleted).
(B) In PC1 vs. PC3, EE% and recovery strongly correlated, independently of the encapsulation process; this is what was already observed in Figure 6C.
(C) Although less relevant overall (only 41.5% of explained variance), the analysis of PC2 vs. PC3 confirmed that the nature of the encapsulation process, of the pre-treatment, and of the hydrophobe are completely independent of each other.
(D) In none of these combinations of PCs do there seem to be correlations between EE% and LogP, although this is probably due to the small range of hydrophobes adopted in this study, as well as the different molecular structures (cyclic or linear).

Finally, it is worth analyzing scores; as we are mostly interested in EE% and recovery, we here focus on the corresponding score space, where experimental points were color-coded according to the encapsulation process (Figure 7C). Please note that if the data points are in the same loading space (i.e., they are correlated) the EE%/recovery is high; if loading spaces are opposite with respect to the center (=inverse correlated), EE%/recovery is low, while positioning at 90° to the center (=not correlated) corresponds to random EE%/recovery. In PC1 vs. PC2, only samples undergoing encapsulation in acetone or oil showed high EE%, although they mostly refer to linalool or 1,6-dihydrocarvone, and depleted cells have low EE% also in these cases; the landscape for recovery was substantially identical. In PC1 vs. PC3, both EE% and recovery are highest for acetone and oil encapsulation of linalool and 1,6-dihydrocarvone, with limonene displaying erratic behavior, and depleted cells found again at low EE%/recovery (permeability inversely correlated to EE% in this PC combination). PC2 vs. PC3 was less informative, as it is dominated by the encapsulation process and for this reason it clearly distinguishes only between oil, water, and acetone processes.

### 2.5. Encapsulation of Hydrophobe Combinations

**Figure 8:**
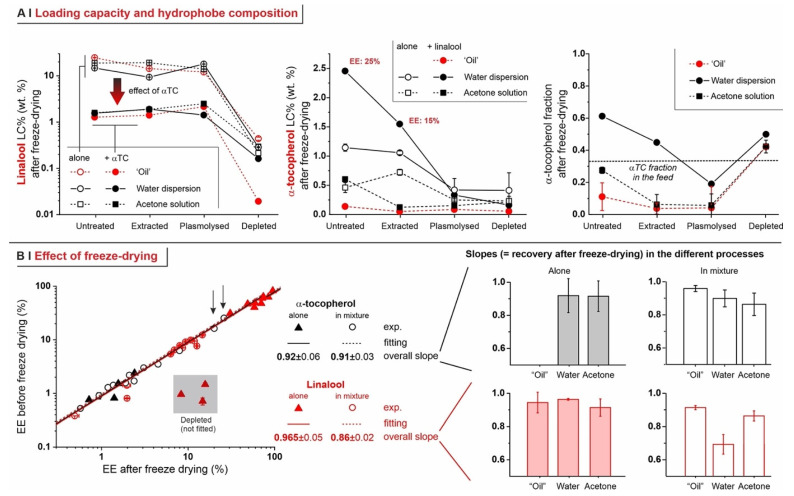
(**A**) Loading capacity of linalool (**left**) and α-tocopherol (**middle**) in the four differently treated yeasts after freeze-drying, compared to their respective controls (hydrophobe alone or diluted in acetone). Please note that the corresponding data before freeze-drying are reported in Figure S11. The fraction of α-tocopherol in the hydrophobe mixture (**right**) generally differs from that in the feed (0.33, as indicated by the grey dashed line); untreated and extracted yeast in water showed a clear enrichment in α-tocopherol, whereas the data for depleted cells should not be considered due to low encapsulation of both hydrophobes. The individual fractions of the two hydrophobes before and after freeze-drying are reported in the Supporting Information, Figure S11B. *n* = 3 instrumental replicates. (**B**) (**Left**) Hydrophobe retention after freeze-drying; black arrows indicate the samples with the highest α-tocopherol loadings (and highest EE, see red labels in panel (**B**). (**Middle**,**right**) Linalool (red symbols and bars in the lower part of the panel) was retained almost quantitatively except when in a mixture, where the average 86% recovery (slope 0.86) was, however, almost solely due to the water-based encapsulation (slope 0.69). α-tocopherol (black symbols and bars) was retained almost completely in all cases, with slopes showing a 90+% recovery. All linear fittings are shown in the Supporting Information, Figure S12.

The use of acetone solutions appears to be advantageous, because this process had a rather high encapsulation efficiency regardless of the nature (and polarity) of the hydrophobe; yet, handling organic solvents may not be possible or advisable in many applications, e.g., in food. However, the underlying concept, i.e., the use of a ‘carrier’ (in this case acetone, but in the past we demonstrated it also for DMSO [37]) for a compound that does not encapsulate well, in principle can be extended to any substance that is liquid, diffuses well into yeast cells, and is easily miscible with the compound of interest (hereafter called the ‘target’). The place of acetone may therefore be taken by, e.g., a terpene. The side advantage is the addition of a function (e.g., fragrancy), but a possible risk is that the carrier and target may interfere, e.g., by competing for the same lipid pockets. Here, we have studied whether linalool (considered as the carrier) might be used in combination with α-tocopherol (vitamin E, here considered as the target); the encapsulation of the latter as an ‘oil’ is impossible due to high viscosity, and, although it can work from acetone or water, the use of linalool is advantageous in the absence of organic solvents and in the possibility of a double functionality (e.g., antioxidant through α-tocopherol also known as vitamin E + scent of linalool). In this binary encapsulation, linalool loaded similarly to when it was used alone (untreated ~ extracted ~ plasmolyzed >> depleted, independently of the process), but the LC% was strongly reduced: the presence of α-tocopherol lowered linalool encapsulation by about one order of magnitude (Figure 8A, left).

α-tocopherol encapsulated in reasonable amounts (LC > 1%) only when used in water dispersion, and in untreated or extracted cells; in these cases, the presence of linalool significantly increased its loading (Figure 7A, middle) and even enriched, in α-tocopherol, the composition of the hydrophobic mixture (originally 67% in linalool; Figure 8A, right). We are inclined to interpret α-tocopherol’s loading performance in relation the viscosity of its dispersions: viscosity would decrease (and therefore dispersibility and likely encapsulation increase) at the higher temperature of the water process and even more so upon dilution with linalool. On the other hand, the high viscosity of ‘oils’ even with linalool (mainly because of low temperature) would be detrimental. Last, the intermediate performance of acetone solutions can also be explained through their intermediate viscosity, which is also substantially unaffected by the presence of linalool.

The encapsulation of linalool was more puzzling, since it appeared to be always hindered, and this happened independently of the amount of α-tocopherol loaded. Interestingly, linalool showed a low recovery upon freeze-drying, indicating its presence on or between yeast cells, only in the water-based encapsulation (Figure 8B), i.e., when α-tocopherol encapsulated the highest. We have therefore interpreted linalool’s much lower encapsulation in the presence of α-tocopherol, as the latter possibly ‘clogging’ the cell walls/surfaces.

In summary, it would appear that the concept of using a carrier/target hydrophobe combination can be advantageous, but only if viscosity is sufficiently low and if the gain of the higher encapsulation of a more precious but ‘difficult’ compound offsets a potentially lower encapsulation in the carrier.

## 3. Conclusions

This study has focused on the understanding of some basic aspects of microencapsulation in yeast, doing so first by developing appropriate companion analytics and then establishing structure–activity relations above all to link.

(1) Analytics. We have developed methods that quantitatively account for fractions of material, such as those adsorbed on plasticware or on glassware, which are often overlooked in the literature, but are absolutely not negligible (see Figure 4C). This higher accuracy allows for a considerably more reliable assessment of the ‘real’ encapsulation (based on the loss during freeze-drying), as opposed to adsorption or physical entrapment. For example, we have demonstrated that maintaining the yeast cytoplasmic content of yeast cells is crucial to minimize the volatility of encapsulated hydrophobes; indeed, they easily evaporate or diffuse out of depleted cells. This is remarkable, since several studies have been based on the assumption that empty capsules could contain more hydrophobes [7].
(2) Effects of yeast pre-treatments. A major point that we have addressed is whether pre-treating yeast may or may not lead to increased levels of encapsulated hydrophobes, a point where the literature reports are fragmented and occasionally contradictory. Plasmolyzed cells are sometimes reported as better performing [11], linking this to the extraction of proteins/nucleic acids at a temperature higher than the membrane transition temperature (45 °C > 37.5 °C) [38], but, all-in-all, the evidence is scant and the overall landscape unclear. Using conditioned yeast (washing and freeze-drying; removal of excipients without damaging cell structure; see Figure 1B), we have shown that yeast depletion—a saponification at high pH and temperature—was the most aggressive pre-treatment and modified both the morphology and composition of the yeast cells: they were reduced to their mere cell walls and the permeability through this last residual barrier was much larger than that through the original wall/membrane combination. The other two pre-treatments, i.e., plasmolysis and ethanol extraction, did not significantly alter cell morphology, nor lipid or protein levels (Figure 2A,B), but moderately increased permeability (increased Nile Red diffusion coefficient) and periplasmic thickness. Physico-chemically, these two pre-treatments are very different: one uses a hypertonic saline solution, the other a rather hydrophobic organic solvent; yet, they may have a similar, denaturing effect on proteins. Thus, it is tempting to ascribe the higher permeability and thickness to the denaturation of lipid-associated proteins causing alterations in the structure and anchoring of the cell membranes. If we add the encapsulation results to the above observations, it may be tempting to draft a direct relation between encapsulation efficiency, resistance to evaporation, and lipid (or protein) levels: they are all very low in depleted yeast, and rather similar in untreated, plasmolyzed, and extracted cells. However, the situation is more complex: analyzing together all processes and actives (Figure 6B), encapsulation seemed not only to massively decrease upon depletion, but to possibly already falter upon plasmolysis/ethanol extraction, which is a trend opposite to what is seen for permeability (Figure 2A,B and Figures S5). There appears, therefore, to be an inverse relation between the speed and capacity of encapsulation, which suggests the presence of key components—which we surmise to be membrane proteins—acting as gatekeepers of hydrophobe loading. Therefore, a tentative interpretation is that protein denaturation caused by plasmolysis/ethanol extraction mildly increases permeability and decreases encapsulation, possibly via an altered organization of lipid-based barrier structures (i.e., the cell membrane), while removal of lipids (and proteins) in the more aggressive conditions of yeast depletion, produces more drastic effects, including a much higher volatility.
(3) Effects of the process. In single-compound encapsulation experiments performed without a carrier (=no solvent, i.e., ‘oil’ or water dispersion), logP appeared to negatively correlate with EE%. It seems reasonable to link this effect to the hydrophobe’s solubility in the cell walls, which we have previously described as a major determinant of encapsulation [37] and would indeed decrease with increasing logP. With acetone solutions, perhaps not surprisingly, the encapsulation efficiency was not significantly affected by the nature of the hydrophobe. This prompted us to try double-compound encapsulation, where a first hydrophobe would act as a ‘carrier’ for one more difficult to encapsulate on its own. Using a linalool/α-tocopherol mixture, the former is safe and perfumed, and diffuses easily into yeast; the second is usable as a ‘drug’. However, this approach does not appear to hold a general validity and should be assessed on a case-by-case basis, since results are difficult to predict: in our case, α-tocopherol benefitted from the double-compound process only under those conditions (water dispersion; untreated yeast or extracted with ethanol) where it appreciably encapsulated on its own. Further, this effect was, in part, counterbalanced by α-tocopherol hindering the linalool’s entrance.
(4) Additional noteworthy observations:
  (A) Cell wall pre-hydration appears to be beneficial to achieve high EE%, but only when encapsulation is not performed in a water environment.
  (B) The practice of repeating encapsulation cycles has often been used in the literature, based on the belief that it would increase the final loading of the yeast [7]. In our study, however, even when evaporation was completely avoided (5 °C, sealed Eppendorf tubes), we have not observed any significant improvement in loading, whereas, clearly, the overall efficiency of the process was much decreased. Therefore, it seems to us that a strong *caveat* should be issued in relation to this practice.

Last, it is worth pointing out in which directions we believe this study may be developed: (1) a relatively low number of hydrophobes (four) was used, casting also a limit to the range of logP, while a broader variety of chemical architectures would make it possible to highlight more precise relationships; and (b) appropriately genetically modified yeast may allow the identification of the key molecular determinants of hydrophobe encapsulation (membrane proteins?).

## 4. Materials and Methods

### 4.1. Materials

Commercially available dry baking yeast *S. cerevisiae* (Belbake dried yeast, Niemcy, Germany; batch L399854, according to the supplier stabilized with sorbitan monostearate) was purchased from a local store (LIDL, Cornigliano, Genova, Italy). Sodium dodecyl sulfate (SDS), ethanol, HPLC grade methanol, isopropyl alcohol, acetone, anhydrous sodium hydroxide, trifluoroacetic acid (Pour LC-MS), (R)-(+)-limonene, linalool, D-α-tocopherol, and 1,6-dihydrocarvone (mixture of isomers) were purchased from Sigma (Merck KGaA, Milan, Italy); sodium chloride and Nile Red were obtained from Fluorochem (Hadfield, UK), 2 N hydrochloric acid came from Alfa Aesar (Heysham, UK). Concanavalin A-Alexa Fluor 488 was supplied by Thermo Fisher Scientific (Wien, Austria).

### 4.2. Preparative Procedures

Standard conditions for freeze-drying, centrifugation, and cell counting are reported in the Supporting Information, Sections S1 and S2.

*Yeast conditioning.* In this phase, additives potentially present in commercially available samples were removed by washing them with water alone or with a water solution of SDS in order to reduce variability during encapsulation. An amount of 200 mg of dry yeast was dispersed in 4 mL of Milli-Q water and centrifuged, repeating the procedure and then dispersing it in 4 mL of a 10 mM (0.29% wt.) SDS solution, or in 4 mL of Milli-Q water. The samples were centrifuged, discarding the supernatant, and dispersed again in Milli-Q water, repeating the procedure twice before centrifuging and discarding the supernatant one last time. Cell integrity was assessed via SEM, TEM, and confocal microscopy, using three different drying protocols (incubator at room pressure, 40 °C, 24 h; vacuum oven at 0.01 mbar, 40 °C, 24 h; freeze-drying). Representative pictures of the pellets and microscopy imaging of the cells are provided in the Supporting Information, Figures S1 and S2, respectively. In all pre-treatments, lots of 4 g of yeast in 50 mL Falcon tubes were conditioned as described above and used without drying.

*Yeast pre-treatment.* Please note that when the mass of a wet pellet of differently pre-treated yeast is measured, the corresponding dry mass can be calculated using the calibration reported in the Supporting Information, Figure S3A. Pre-treatments were typically applied on 8 g of yeast (two lots of SDS-conditioned yeast).

(A) Cells untreated (control): wet pellets were dispersed in Milli-Q water and centrifuged, discarding the supernatant, three more times.
(B) Cells extracted with ethanol: wet pellets were transferred into a 250 mL round-bottom flask, re-suspended in 80 mL of 50% v. ethanol/water and left at room temperature under stirring at 300 rpm for 2 h. Yeast cells were centrifuged and then washed thrice with Milli-Q water.
(C) Cells plasmolyzed with hypertonic sodium chloride: wet pellets were suspended in 80 mL of 0.9% wt. sodium chloride and centrifuged discarding the supernatant three times, followed by a re-suspension in 80 mL of 20% wt. sodium chloride; the dispersion was transferred into a 250 mL round-bottom flask, left at 45 °C under 300 rpm magnetic stirring for 2 h, centrifuged, and washed once with 40 mL of 0.9% wt. sodium chloride and thrice with Milli-Q water each time after centrifugation.
(D) Cells depleted and reduced to cell walls: wet pellets were resuspended in 80 mL of 1M sodium hydroxide, transferred into a 250 mL round-bottom flask equipped with a condenser, and left at 85 °C under 300 rpm stirring for 1 h. Yeast was centrifuged, discarding the supernatant, and re-suspended in 60 mL of Milli-Q water, transferred back to a clean 250 mL round-bottom flask equipped with a condenser; the pH was adjusted to 4.5 with a 0.5 M hydrochloric acid solution (final volume 80 mL) and the temperature was risen to 60 °C for 1 h, while keeping the dispersion under 300 rpm stirring. Yeast was centrifuged, washed once with Milli-Q water, twice with isopropanol, then with acetone and finally with Milli-Q water three times (always after centrifugation). It was then freeze-dried as reported in the Supporting Information, Section S1. The techniques used to characterize the differently pre-treated yeast cells are reported in the Supporting Information, Section S3.

*Encapsulation procedures.* Linalool (density: 0.858 g/mL; logP: 3.0 [39]), 1,6-dihydrocarvone (density 0.929 g/mL; logP: 3.1 [40]), limonene (density: 0.841 g/mL; logP: 4.4 [39]), a 1:2 wt. α-tocopherol/linalool mixture (measured density: 0.871 g/mL, see Supporting Information, Figure S10A. α-tocopherol’s logP calculated via XLogP3: 3.0; https://pubchem.ncbi.nlm.nih.gov/compound/Tocopherol (accessed on 15 January 2024: 10.7) and a 1:2 wt. α-tocopherol/linalool mixture (measured density: 0.80 g/mL, see Supporting Information, Figure S10B) were used as hydrophobes for all encapsulation protocols.

(A) Encapsulation from an ‘oil phase’ (=no solvent or dispersant). For a comparison between wet and dry yeast, 50 mg of conditioned but untreated yeast (control) was used either directly in a dry form, or as a wet pellet (suspended in 1 mL of Milli-Q Water and centrifuged discarding the supernatant, to yield roughly 210–220 mg of wet material) and exposed to linalool. In detail, yeast (wet or dry) was introduced into 2 mL Eppendorf tubes, and linalool was directly pipetted on top of it in an amount corresponding to 30% wt. of the yeast’s dry weight, vortexing immediately for 30 s; Eppendorf tubes were sealed with Parafilm and incubated in an orbital shaker (5 °C, 54 rpm, 2 h). Since dry yeast showed higher encapsulation (Figure S6, symbols in the bottom row), all further screening experiments of ‘oil’ encapsulation, e.g., with different hydrophobes, only used dry yeast.
(B) Encapsulation from water dispersion. One g of freeze-dried pre-treated yeast was dispersed in 10 mL of Milli-Q water, gently vortexing to ensure homogeneity. An amount of 500 μL aliquots (corresponding to 50 mg of dry yeast) was transferred into 2 mL Eppendorf tubes, centrifuged, discarding the supernatant, diluted in 500-x μL of Milli-Q water, and finally topped up with x μL of hydrophobe (x is the volume corresponding to 30% wt./15 mg of hydrophobe through the density values reported above). The slurries were vortexed again, sealed with Parafilm and incubated in a Thermomixer C (Eppendorf AG, Hamburg, Germany) at 60 °C, 900 rpm for 4 h. In order to estimate the possible loss of hydrophobe due to evaporation, a 2 mL Eppendorf tube containing the same amounts of water and hydrophobes, but no yeast, was incubated as the yeast-containing samples and analyzed.
(C) Encapsulation from organic solution. (1) Choice of the encapsulation conditions. These experiments were conducted with linalool as a probe at 30% wt. in respect to the mass of dry yeast. Fifty mg of conditioned but untreated yeast (control) was used directly in a dry form, or as a wet pellet (suspended in 1 mL of Milli-Q Water and centrifuged discarding the supernatant, to yield roughly 210–220 mg of wet material); the yeast suspension was treated with additional variable volumes (500, 250, 125, or 62.5 µL) of liquid phase (water, ethanol, acetone, 50% *v*/*v* water/ethanol, and 50% *v*/*v* water/acetone) always containing 15 mg of linalool and incubated in an orbital shaker (5 °C, 54 rpm) for 2 h. In experiments featuring repeated encapsulation cycles, the yeast was centrifuged, discarding the supernatant, air dried for 24 h, subjected to the same conditions of encapsulation for a total of four additional cycles of encapsulation (each cycle with 15 mg of linalool) and washing. (2) Encapsulation in acetone. As a result of the screening in point 1 (see Supporting Information, Figure S6), the optimized protocol for encapsulation in organic solution resulted in the following procedure. Fifty mg of freeze-dried yeast in the form of a wet pellet was dispersed into 125 µL of a 120 mg/mL solution of hydrophobe in acetone (barely enough to cover the whole pellet), quickly vortexed, sealed with Parafilm, and incubated in an orbital shaker (5 °C, 54 rpm, 2 h).

*Quantification of hydrophobe encapsulation.* For a rapid overview of the process, please refer to Figure 4B. After encapsulation, yeast dispersions were centrifuged, and the supernatant was analyzed via HPLC. The pellets were washed twice with Milli-Q water, and, after each washing, the yeast suspension was transferred into a new Eppendorf tube for the quantification of a non-encapsulated hydrophobe possibly adhering to the plastic. At the end of this washing phase, the wet pellet was split into two 2 mL Eppendorf tubes, and one portion of them was subjected to freeze-drying. An amount of 180 mg of 0.25–0.50 mm glass beads was added to both wet and freeze-dried samples, suspending them in 500 µL of HPLC-grade methanol, finally proceeding to mechanical disruption (lysis) in a MM 400 bead mill (Retsch, Haan, Germany) operated at 30 Hz for 1 h. After centrifugation, the supernatants were removed; an additional 500 µL of methanol was added to extract the pellets (incubation at 40 °C, 300 rpm for 3 min orbital shaking and centrifugation; fraction called bead wash). All supernatants and washings were then analyzed via HPLC. The amount of encapsulated hydrophobe $m_{H}$ is then calculated as the sum of two contributions, i.e., that in cell lysates $m_{Hlys}$ and that in bead washes $m_{Hbead}$, $m_{H}={m_{Hlys}+m}_{Hbead}=\frac{{n_{lys}\times A_{lys}+n}_{bead}\times A_{bead}}{slope}$, where $n_{lys}$ and $n_{bead}$ are the respective dilutions required prior to HPLC analysis, $A_{lys}$ and $A_{bead}$ are the respective peak absorbance values and slope is the slope of the calibration. $m_{H}$ was then used to calculate encapsulation efficiency (EE%) and loading capacity (LC%) for all processes; the two parameters are, respectively, defined as $m_{H}/m_{H}^{TOT}$ and as the $m_{H}/m_{yeast}^{DRY}$ ratio (both in %).

*Principal Component Analysis (PCA).* The results of the encapsulation experiments, as described in Figure 6A, were subjected to PCA associating numerical values to both input variables (hydrophobe identity—their LogP (linalool: 3, 1,6-dihydrocarvone: 3.1 and limonene: 4); pre-treatments—their D_app_ (as calculated in Figure 3B: untreated: 3.1 × 10^−3^, extracted: 4.1 × 10^−3^, plasmolyzed: 4.2 × 10^−3^, depleted: 6.2 × 10^−3^ s^−1/2^); encapsulation processes—the dielectric constant of the medium (ε of water: 66.7, acetone: 19.5, terpenes, in average: 2.5 [41]) and output variables (encapsulation efficiency and recovery after freeze-drying, both in weight %); see the Supporting Information, Table S7. PCA analysis was then performed using the CAT (Chemometric Agile Tool) software, freely downloadable from https://gruppochemiometria.it/index.php/software (accessed on 4 December 2023).

*Statistical analysis.* Data were processed using the Origin Pro software, version 2018 64bit. Error bars were computed as standard deviations (*n* is specified in the text for each experiment). In detail, Figure 1A (IR spectra): *n* = 24 scans; Figure 2B (IR spectra): *n* = 24; Figure 2C (TEM analysis of morphological indexes): *n* = 40; Figure 3 (spectroscopic measurement of Nile Red diffusion): technical triplicate, *n* = 3; Figure 4C (fractions of linalool recovered from each encapsulation step): experimental triplicate, *n* = 3; Figure 5B (quantification of Nile Red^+^ area at CLSM): *n* > 13; Figure 6 (encapsulation of hydrophobes): instrumental triplicate, *n* = 3; Figure 7 (encapsulation of linalool and α-tocopherol): instrumental triplicate, *n* = 3. Number of replicates referring to data reported only in the Supporting Information can be recovered from the relative captions or from the experimental procedure. Errors of calculated values were derived from statistic error propagation. Student tests were performed at a 95% of confidence interval. One Way ANOVA tests were carried out at three levels of confidence with both a Bonferroni and a Tukey post hoc test.

### 4.3. Characterization Techniques

*High performance liquid chromatography (HPLC).* Samples in water or methanol from yeast extraction or centrifugation were diluted with methanol in order to fit within the linear HPLC calibration for the various hydrophobes. The diluted samples were filtered through 0.22 μm PTFE syringe filters; their HPLC analysis was carried out with an Agilent 1260 Infinity II HPLC (Agilent Technologies, Santa Clara, CA, USA) equipped with a quaternary pump, a diode-array detector, an auto-sampler, a column temperature controller, and a Poroshell Eclipse column (150 × 4.6 mm ID, 4 μm; EC-C18), working at 40 °C, with an injected volume of 10 μL, and at a flow of 1 mL/min. Mixtures of water and methanol with 0.1% TFA were used as eluents under non-isocratic conditions for linalool, 1,6-dihydrocarvone (mixture of the two isomers), S-(-)-limonene, and α-tocopherol (see Supporting Information Table S1), the absorbance of which was detected with the diode-array detector at 210 nm (and 290 for samples containing α-tocopherol). Calibrations were performed using dilutions of the hydrophobes as standards. For each sample, *n* = 3, instrumental repeats.

*Infrared spectroscopy (IR).* Dry yeast samples weighing about 2 mg were deposited on and then pressed against the window of an attenuated total reflection infrared spectrometer ALPHA II (Bruker, Milan, Italy). The signal was acquired at room temperature, averaging 24 scans with a resolution of 4 cm^−1^.

*Scanning electron microscopy (SEM).* Yeasts were fixed in a solution of 2% glutaraldehyde in 0.1 M cacodylate buffer for 2 h at room temperature. After several washes in the same buffer, the samples were post-fixed in 1% osmium tetroxide in Milli-Q water for 2 h and washed with Milli-Q water. Yeast was subsequently dehydrated with a series of 10 min incubations in rising concentrations of ethanol in water solutions (from 30 to 100%), 1:1 ethanol: hexamethyldisilazane (HMDS, Sigma-Aldrich, St. Louis, MI, USA), and 100% HMDS and dried overnight in air. The samples were then sputtered with a 10 nm gold layer and analyzed using a JEOL (Tokyo, Japan) JSM-6490LA Scanning Electron Microscope (SEM) equipped with a tungsten filament and operating at 10 kV of accelerating voltage.

*Transmission electron microscopy (TEM).* Yeast samples were hydrated in PBS buffer pH 7.4 for 1 day at RT, followed by fixation in 2% glutaraldehyde in 0.1 M cacodylate buffer for 1 h at room temperature. They were post-fixed in 1% osmium tetroxide in Milli-Q water for 1 h, washed in Milli-Q water and stained overnight at 4 °C in an aqueous 1% uranyl acetate solution. Then, the samples were dehydrated in a graded ethanol series and propylene oxide was used to allow the resin (used in the following steps) to permeabilize the cell wall. After several washings in propylene oxide-EPON resin 3:1, 1:1: and then 1:3, the yeast was embedded in pure EPON resin for 48 h at 65 °C. Sections of about 70 nm were cut with a diamond knife on a Leica EM UC6 ultramicrotome. Transmission electron microscopy (TEM) images were collected with a Jeol JEM 1011 (Jeol, Tokyo, Japan) electron microscope equipped with a 2 Mp charge-coupled device camera (Gatan Orius, Pleasanton, CA, USA).

*Nile Red permeation.* The optimization of the analytical conditions is described in the Supporting Information, Section S4. Please note that Nile Red diffusion in yeast is rather slow (at least a few hours to reach steady state [42]), but also very dependent on the yeast type [43]; thus, a permeation enhancer, such as DMSO, is often used for easier comparative staining [30,44]. As in other examples from the literature [5,45], here we have used low amounts of DMSO as a compromise between reasonably rapid kinetics and a sufficient sensitivity to cell barriers. An amount of 225 μL of a 1 × 10^7^ cells/mL dispersion of yeast in Milli-Q water was introduced in each well of a 96-well plate, confirming their concentrations as described in the Supporting Information, Section S3, cell counting B. Separately, a 11.7 μg/mL Nile Red dispersion was prepared by diluting 1:85 *v*/*v* a stock 1 mg/mL DMSO with Milli-Q water; 33 μL of the resulting dispersion was added to each well, ensuring good mixing by pipetting vigorously. Of note, Nile Red diffusion in yeast is rather slow, a few hours may be required to follow the process to completion [42], and its rates may sharply depend on the yeast type [43]; thus, DMSO is often used to speed up permeation and thus allow a rapid comparative staining [30,44]. However, low amounts of DMSO can also allow Nile Red permeation in reasonably short times [5,45], and here we used an overall 1.9% concentration of DMSO as a compromise between rapidity of the permeation (higher with larger DMSO content) and differences between samples (larger with smaller DMSO content). The development of fluorescence (ex./em. 545 nm/620 nm) was recorded at 37 °C, shaking the plate at 425 rpm (double orbital shaking, 3 mm) for 30 s before each reading; time points were recorded every 1.5 min for 24 min, then every 15 min for an additional 60 min, and finally every 60 min for the following 10 h. At the end of the experiment, the fluorescence readings of each well were compared to that of non-monitored wells to assess the degree of Nile Red bleaching, which was always found to be negligible. The data were fitted with a single exponential model $I\left(t\right)=I_{0}+A\left(1-e^{-\frac{t}{\tau }}\right)$ where $I\left(t\right)$ is the time-dependent intensity of emission at 620 nm, t is the time, and the parameters $I_{0}$ (representing the emission intensity at time zero), A (summed to $I_{0}$ provides the asympotic emission intensity), and τ(the characteristic time of the permeation process) were tunable. Under conditions of Fickian diffusion, $I\left(t\right)$ depends linearly on $\sqrt{t}$, and the slope is proportional to the asymptotic emission (which depends on the water/yeast partition coefficient of Nile Red), to the apparent diffusion coefficient of the fluorophore, and to geometrical factors (shape of the yeast, thickness and number of the barrier structures). Therefore, the slope of a graph $I\left(t\right)/\left(I_{0}+A\right)$ vs. $\sqrt{t}$ is linearly proportional to Nile Red’s apparent diffusion coefficient D_app_. All numerical data (experimental or fitted) are provided in the Supporting Information, Tables S2–S4.

*Confocal laser scanning microscopy (CLSM).* Ten mg of yeast was suspended in 948 μL of Milli-Q Water. Lipid bodies and cell walls were, respectively, stained by the addition of 2.5 μL of a 1 mg/mL Nile Red solution in DMSO, and of 50 μL of a 50 μg/mL solution of Concanavalin A-Alexa Fluor 488 in 0.1 M of NaHCO_3_ and 2 mM NaN3. The mixture was incubated for 30 min in the dark at 37 °C, 600 rpm, then centrifuged and the supernatant discarded; the pellet was washed once with Milli-Q Water and at last suspended in 3 mL of water. An amount of 2.5 µL of the sample was placed on a microscopy glass and sealed with a glass lead. Confocal analysis was carried out with a SP5-inverted microscope (Leica, Milan, Italy). Samples were observed through a 63× (1.4 NA), oil objective, zoom level 7, using lasers at 488 and 564 nm, respectively, for Concanavalin A-Alexa Fluor 488 and Nile Red (all acquisition parameters kept constant) and analyzing the pictures with ImageJ (Fiji) in two different ways. (a) Qualitatively: the signal was adjusted in brightness and contrast to compensate for Nile Red bleaching during acquisition. (b) Semi-quantitatively: the number of non-black pixels in the Nile Red channel was recorded after the application of an automatic threshold without image treatment. Cell area and perimeter were measured with manual contour tracing and ImageJ was employed for the automatic recognition of the minor and major axes and the ensuing calculation of the cell roundness (defined as the length ratio between the two).

## Data Availability

The datasets generated during and/or analyzed during the current study are available from the corresponding author on reasonable request.

## Supplementary Materials

The following supporting information can be downloaded at: https://www.mdpi.com/article/10.3390/molecules29020539/s1.
