# Supplementary material for: A Quantitative Re-Assessment of Microencapsulation in (Pre-Treated) Yeast
Source: Molecules. 2024 Jan 22;29(2):539. doi: 10.3390/molecules29020539 (PMC10818300; doi:10.3390/molecules29020539)
Supplement: Supplementary file 1 [file molecules-29-00539-s001.zip › molecules-2668966-supplementary.pdf]

A quantitative re-assessment of microencapsulation in (pre-treated) yeast.

Giulia Coradello [1,2], Chiara Setti [1,§], Roberto Donno [1,$], Matilde Ghibaudi [1,¶], Federico Catalano [3] and Nicola Tirelli [1,*]

[1] Laboratory for Polymers and Biomaterials, Fondazione Istituto Italiano di Tecnologia, 16163 Genova, Italy; giulia.coradello@iit.it, nicola.tirelli@iit.it
[2] Department of Chemistry and Industrial Chemistry, University of Genoa, Via Dodecaneso 31, 16146 Genova, Italy; giulia.coradello@iit.it
[3] Electron Microscopy Facility, Istituto Italiano di Tecnologia, via Morego, 30, 16163 Genoa, Italy; federico.catalano@iit.it

[§] Current address: Department of Experimental Medicine (DIMES), University of Genoa, via G.B. Marsano 10, 16132 Genova, Italy; settichiara.90@gmail.com
[$] Currently: Independent researcher; robertodonno@gmail.com
[¶] Current address: Institute for Bioengineering of Catalonia (IBEC), Carrer de Baldiri Reixac, 10, 12, 08028 Barcelona, Spain; mghibaudi@ibecbarcelona.eu,

* Correspondence: nicola.tirelli@iit.it

Supporting information

S1. Materials and methods

Table S1. Elution conditions for the HPLC analysis of hydrophobic actives.

Time (min)	Vol % water phase [a]	Time (min)	Vol % water phase
Linalool (λ = 210 nm; elution time: 14.8 min)		*Limonene* (λ = 210 nm; elution time: 20.2 min)	
0 - 2	60	0 - 3	60
2 - 6	60 → 40	3 - 11	60 → 20
6 - 9	40	11 - 16	20
9 - 17	40 → 20	16 - 21	20 → 0
17 - 22	20	21 - 26	0
22 - 25	20 → 0	*α-tocopherol/linalool or acetone 1:2 wt.* (λ = 290 and 210 nm resp.; elution times 16.1 and 3.2 min resp.)	
25 - 30	0 → 40		
30 - 33	40	0 - 2	75 → 85
Dihydrocarvone (λ = 210 nm; elution time: 6.6 min)		2 - 13	85 → 100
0 - 5	40	13 - 16	100
5 - 13	40 → 20	16 - 26	100 →0
13 - 16	20 → 0	26 - 33	0 → 75
16 - 19	0	33 - 38	75
19 - 22	0 → 40		

[a] water phase: 0.1% TFA in Milli-Q water. The methanol phase (0.1% TFA methanol) is the complement to 100%.

Centrifugation. Samples in 2 mL Eppendorf tubes were subjected to centrifuge force using a Centrisart G-16C centrifuge (Sartorius, Varedo, Italy), those in 50 mL Falcon tubes using an A-16C centrifuge (Sartorius, Varedo, Italy), both operated at 7000 g and 25°C, always for 3 min.
Freeze drying. Yeast suspensions in MilliQ water (in 2 mL Eppendorf or 50 mL Falcon tubes) were frozen in liquid nitrogen (-196°C) and inserted into the cold chamber (-80°C) of an Epsilon 2-4 LSCPlus freeze dryer (Martin Christ Gefriertrocknungsanlagen GmbH, Osterode am Harz, Germany). A standard drying program for water was then applied, with an overall duration of 16 h, and final shelf temperature of 10°C and pressure of 1.14 mbar; the dried samples were stored at 4-6°C in Parafilm-sealed Eppendorf tubes.

S2. Characterization of yeast following conditioning

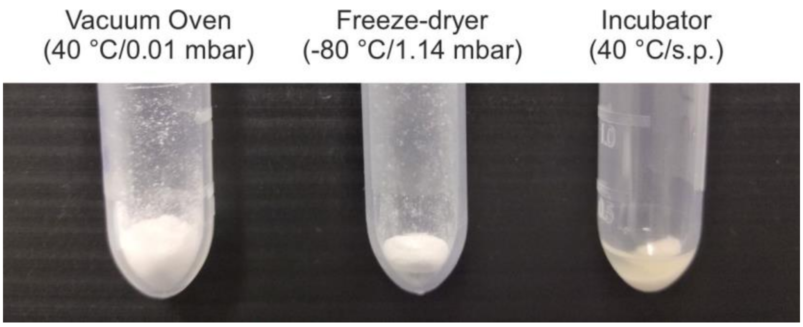

Figure S1. Yeast samples pre-conditioned only with Milli-Q water, dispersed in 2 mL Eppendorf tubes and dried through different procedures. It is noticeable that the use of an incubator at room pressure (Eppendorf tube on the right) did not allow the complete evaporation of water.

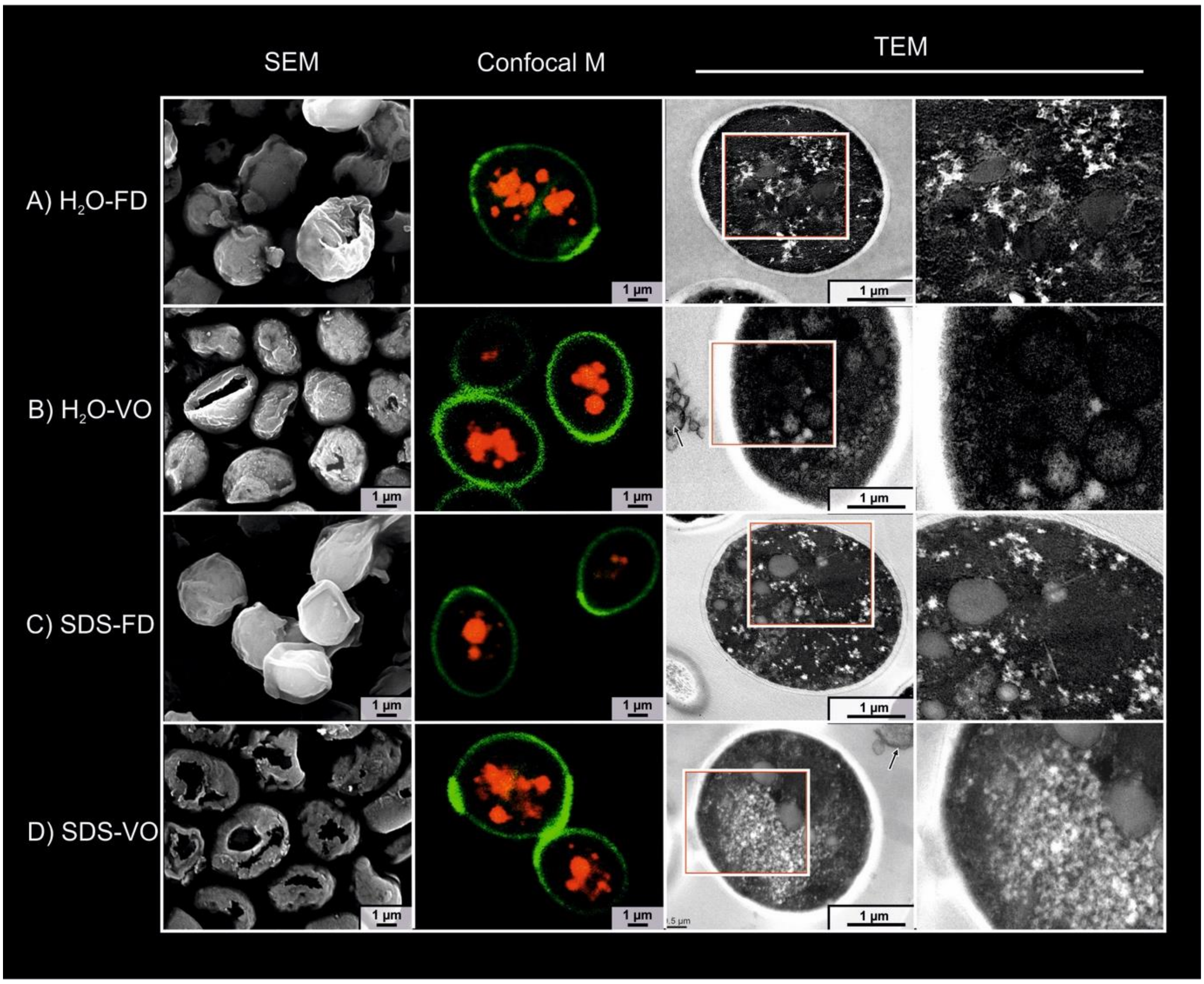

Figure S2. Yeast cells were conditioned *via* repeating washing with Milli-Q water alone (H_2O) or with water and SDS (SDS), and dried in a vacuum oven (VO) or freeze dried (FD). Although confocal microscopy (red: apolar regions stained with Nile Red; green: cell wall mannans stained with Concanavalin A-Alexa Fluo 488) and TEM did not show significant differences between protocols, it is apparent *via* SEM that oven drying produced significant damages to the cell walls.

S3. Characterization of yeast following pre-treatment

Wet-dry mass correlation for yeast pellets. A calibration was performed to calculate the dry mass of wet pellets, which is effectively a measure of 'water absorption capacity' of the yeast samples; wet pellets of differently yeast obtained from dispersions in Milli-Q water were weighted (wet weight), freeze-dried, and weighted again (dry weight; Figure S3A).

Yeast cell counting. Cell numbers were measured indirectly, using data from either weight or optical density measurements, which were previously calibrated against cell numbers as described below:

A) Through measurements of the weight of yeast pellets. Freeze-dried pellets were weighted, dispersed in Milli-Q water, and cell numbers were measured using a Bürker chamber according to the procedure reported in Section 1SI (Figure S3B); a correlation to wet masses can be obtained by using the previous wet-dry mass correlation.

B) From measurements of the optical density of yeast dispersions. 225 µL of yeast dispersions at variable concentration in Milli-Q water were added in each well a 96-well plate, measuring their optical density at 600 nm (Sinergy H1 plate reader; Biotek, Swindon, United Kingdom); samples from each well were diluted, and cell numbers measured using a Bürker chamber (see Supporting Information, Figure S3C).

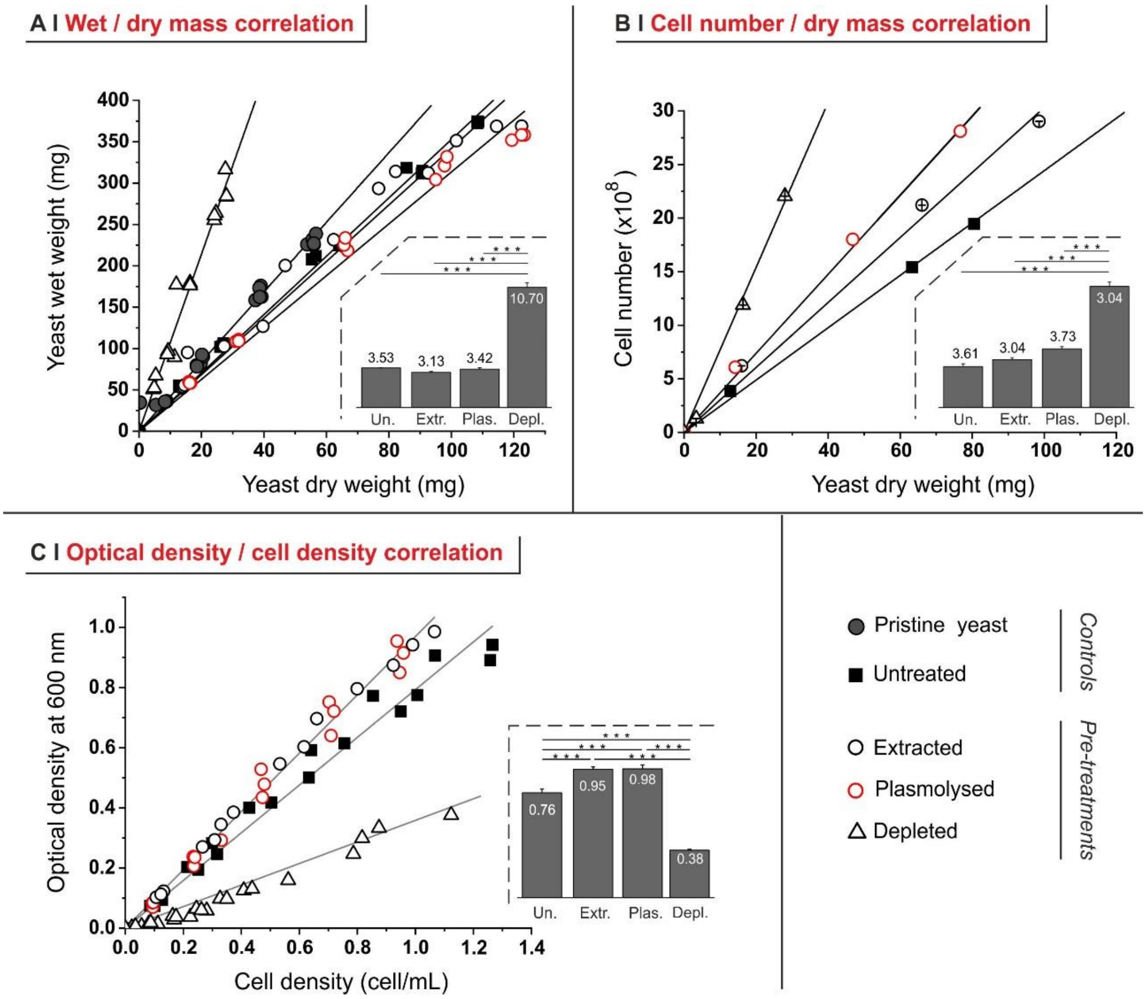

Figure S3. A. Wet *vs*. freeze dried mass of each pre-treated yeasts; the slopes obtained through a linear fitting (reflective of yeast water adsorption capacity) are reported in the inset. **B.** Number of cells *vs*. dry mass linear correlation for all pre-treatments; the slopes (indicating cell weight) are again reported in the inset. **C.** Optical density at 600 nm *vs*. cell concentration; the slopes obtained through a linear fitting are reported in the inset

and they account for differences in composition or dimensions of each pre-treatment cells. In all insets, the stars refer to statistically different results ($p<0.001$) according to a Student t-test.

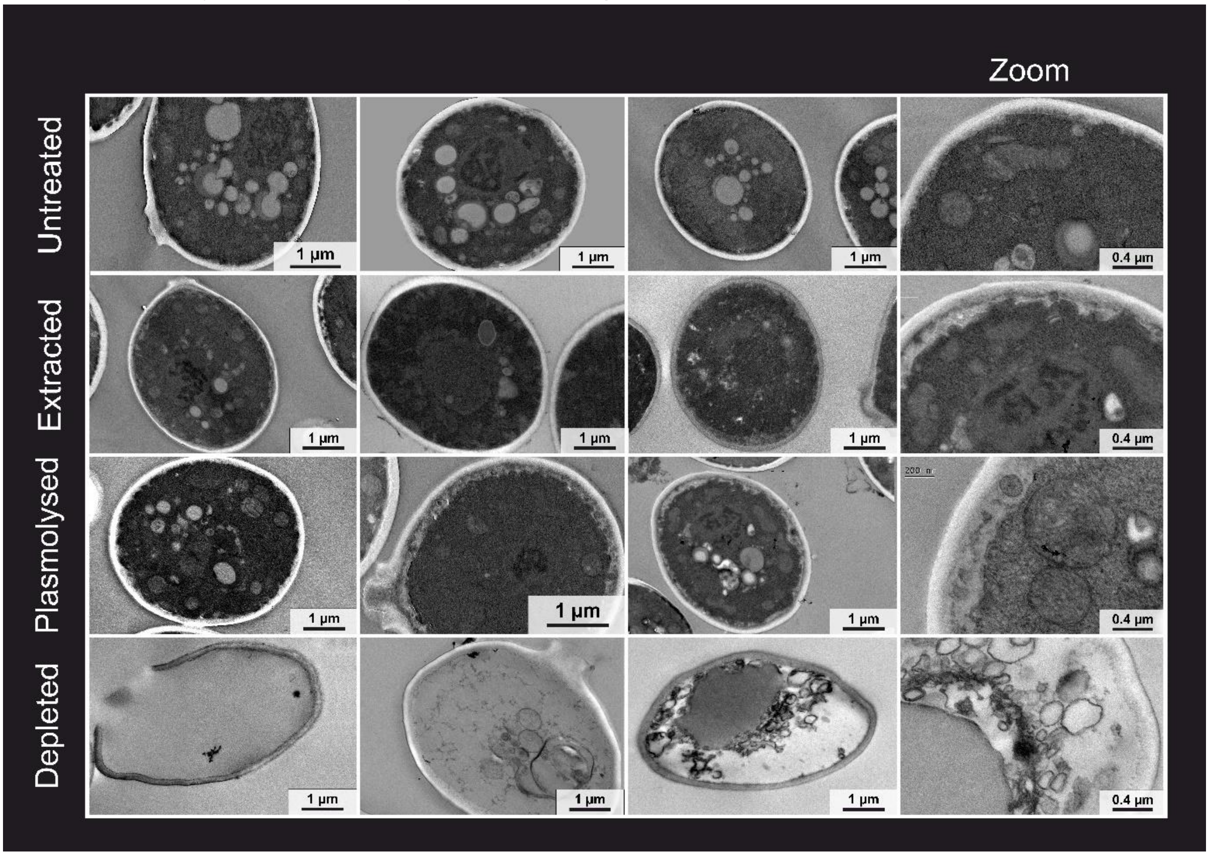

Figure S4. Representative TEM pictures of yeast cells (untreated or after various pre-treatments) are here provided as examples of the reproducibility of the processes.

4SI. Assessment of cell wall permeability using Nile Red diffusion

Please note that Nile Red fluorescence is quenched by water; hence the extracellular fluorescence is always negligible (in any case, it was always employed as a negative control) and the readings are therefore always related to the Nile Red present intracellularly in yeast.

225 µL of 0.25, 0.6 and 1x10^7 cells/mL dispersion of yeast in Milli-Q water were introduced in each well of a 96-well plate, confirming their concentrations as described above (Section 3SI, cell counting, method B). 33 µL of 11.7, 18.8, 46.9 and 78.2 µg/mL Nile Red solutions in Milli-Q water were added, respectively yielding 1.5, 2.4, 6, 10 µg/mL final concentrations of the fluorophore. Emission spectra (535 - 800 nm; excitation at 545 nm) were recorded after 1 hour of incubation at 37°C (Figure S5A). 1.5 µg/mL Nile Red and 10^7 cells/mL were selected for further experiments, since a) higher Nile Red concentrations show significant quenching and thus deviation from a linear relation between emission intensity/concentration (data not shown), b) this cell density minimizes the uncertainty in cell number determination via optical density, while still allowing linearity in Nile Red fluorescence vs. concentration (Figure S5B).

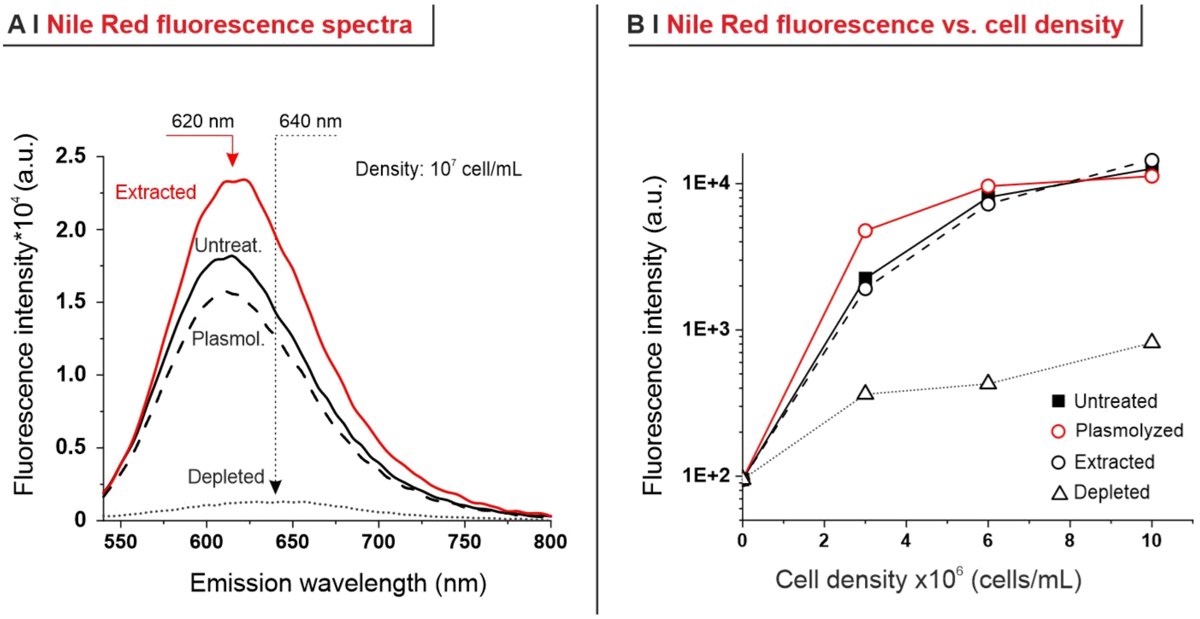

Figure S5. ***A.*** Representative fluorescence spectra of yeast cell dispersions at a 1.5 µg/mL Nile Red concentration, as a function of pre-treatment. ***B.*** Maximum emission intensity (excitation at 620 nm; 1.5 µg/mL Nile Red) as a function of cell density. Please note that for a better comparison between depletion and other pre-treatments, data are presented in a semi-log fashion; however, actually fluorescence intensity scales linearly with concentration.

Table S2. Emission data (arbitrary units) for Nile Red permeation experiments (see Figure 3.A). All data were averages of three independent experiments.

Time (s)	Control yeast		Plasmolyzed yeast		Extracted yeast		Depleted yeast	
	Emission	SD	Emission	SD	Emission	SD	Emission	SD
300	1103	47	1758	38	1224	45	77	70
390	1177	88	1847	22	1374	60	114	96
480	1178	98	1893	18	1460	33	132	124
570	1224	110	1941	78	1507	52	151	114
660	1238	86	2005	37	1487	49	182	131
750	1264	90	2010	43	1514	24	216	126
840	1321	72	2078	59	1587	86	232	150
930	1362	69	2056	62	1614	63	270	168
1020	1404	76	2092	31	1657	53	248	135
1110	1445	86	2086	49	1718	28	266	113
1200	1488	54	2137	45	1750	85	282	168
1290	1555	92	2211	29	1822	59	295	148
1380	1630	74	2297	42	1919	63	303	164
2280	2106	4	2662	87	2389	254	342	160
3180	2700	60	3224	216	2976	296	387	140
4080	3240	93	3929	309	3665	324	408	134
4980	3783	144	4615	253	4220	367	445	158
5070	3843	120	4767	333	4341	412	494	156
8670	5778	309	7150	304	6112	688	675	140
12270	7318	479	8758	297	7453	670	777	204
15870	8450	459	9965	200	8505	612	886	130
19470	9151	415	10621	77	9046	584	981	118
23070	9615	257	11096	100	9416	280	1020	125
26670	9840	177	11220	170	9680	147	1119	109
30270	9944	45	11327	30	9933	293	1120	94
33870	9774	121	11097	134	10061	366	1142	24
37470	9560	149	10809	214	10002	325	1126	21
41070	9404	322	10502	324	9779	258	1177	66
41160	9503	252	10541	277	9935	449	1171	116

Table S3. Normalized fluorescence data (arbitrary units) for the calculation of Nile Red relative apparent diffusion coefficients. Only the data in red were used for the calculation of the slope of the linear region (Fickian diffusion) and the resulting calculation of relative apparent diffusion coefficients.

Time (s)	Control yeast		Plasmolyzed yeast		Extracted yeast		Depleted yeast	
	Emission	SD	Emission	SD	Emission	SD	Emission	SD
17	0.0429	0.0135	0.0799	0.0191	0.0654	0.0189	0.0650	0.0609
20	0.0458	0.0159	0.0839	0.0192	0.0735	0.0218	0.0964	0.0841
22	0.0459	0.0163	0.0860	0.0195	0.0781	0.0215	0.1122	0.1079
24	0.0476	0.0173	0.0882	0.0227	0.0806	0.0232	0.1279	0.0998
26	0.0482	0.0165	0.0912	0.0215	0.0795	0.0227	0.1545	0.1147
27	0.0492	0.0169	0.0914	0.0218	0.0810	0.0218	0.1826	0.1118
29	0.0514	0.0168	0.0945	0.0232	0.0849	0.0260	0.1963	0.1319
30	0.0530	0.0171	0.0934	0.0231	0.0863	0.0252	0.2289	0.1481
32	0.0546	0.0178	0.0951	0.0221	0.0886	0.0253	0.2106	0.1194
33	0.0562	0.0187	0.0948	0.0228	0.0919	0.0247	0.2253	0.1016
35	0.0579	0.0179	0.0971	0.0232	0.0936	0.0282	0.2388	0.1481
36	0.0605	0.0201	0.1005	0.0232	0.0974	0.0278	0.2501	0.1319
37	0.0634	0.0202	0.1044	0.0246	0.1026	0.0293	0.2572	0.1458
48	*0.0820*	*0.0225*	*0.1210*	*0.0302*	*0.1278*	*0.0459*	*0.2895*	*0.1432*
56	*0.1050*	*0.0309*	*0.1466*	*0.0417*	*0.1591*	*0.0561*	*0.3277*	*0.1274*
64	*0.1261*	*0.0380*	*0.1786*	*0.0529*	*0.1960*	*0.0669*	*0.3455*	*0.1227*
71	*0.1472*	*0.0457*	*0.2098*	*0.0571*	*0.2257*	*0.0767*	*0.3769*	*0.1431*
71	*0.1495*	*0.0454*	*0.2167*	*0.0622*	*0.2321*	*0.0807*	*0.4187*	*0.1425*
93	*0.2248*	*0.0732*	*0.3250*	*0.0844*	*0.3269*	*0.1195*	*0.5719*	*0.1336*
111	*0.2847*	*0.0962*	*0.3981*	*0.1000*	*0.3985*	*0.1366*	*0.6588*	*0.1894*
126	*0.3288*	*0.1074*	*0.4530*	*0.1075*	*0.4548*	*0.1478*	*0.7510*	*0.1293*
140	*0.3561*	*0.1132*	*0.4828*	*0.1084*	*0.4837*	*0.1536*	*0.8311*	*0.1209*
152	0.3741	0.1119	0.5043	0.1141	0.5035	0.1424	0.8645	0.1282
163	0.3829	0.1112	0.5100	0.1186	0.5177	0.1388	0.9486	0.1165
174	0.3869	0.1071	0.5149	0.1132	0.5312	0.1500	0.9493	0.1040
184	0.3803	0.1083	0.5044	0.1157	0.5380	0.1557	0.9682	0.0450
194	0.3720	0.1071	0.4913	0.1165	0.5349	0.1527	0.9546	0.0419
203	0.3659	0.1122	0.4774	0.1184	0.5229	0.1461	0.9974	0.0810
203	0.3698	0.1105	0.4791	0.1167	0.5313	0.1584	0.9922	0.1232

Table S4. Numerical values of fitted parameters (single exponential fitting) for the data in Table S2 after single exponential fitting, and for the data in Table S3 (linear fitting).

	y0		A		τ (sec)		Slope (proportional to approximated diffusion coefficients) ($sec^{-1/2}$)	
	Value	SD	Value	SD	Value	SD	Value	SD
Control	838	37	24851	6970	43413	13334	0.00295	0.00087
Plasmolyzed	1562	44	20526	4747	34606	10993	0.00398	0.00094
Extracted	1100	30	17680	4703	29946	9515	0.00408	0.00113
Depleted	109	15	1067	15	11023	898	0.00617	0.00035

S5. Encapsulation experiments

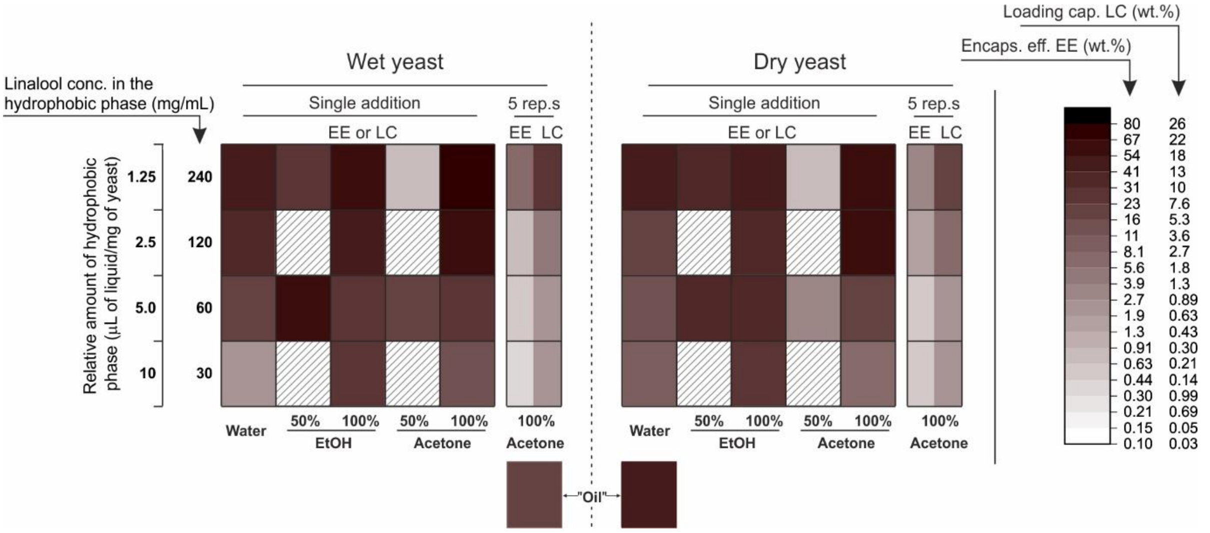

Figure S6. Screening of different solvent compositions for 5°C encapsulation of linalool from organic solution (in acetone or ethanol, or in 50:50 Milli-Q water:acetone/ethanol) and yeast hydration state, using the 60°C encapsulation from water dispersion (left columns, 'water') and the 5°C encapsulation from oil (bottom, 'oil') as controls. Please note that encapsulation efficiency (EE%)/loading capacity (LC%) are color-coded in a non-linear fashion (double log reciprocal), in order to better appreciate differences between their lowest values. In all experiments, 50 mg of dry yeast were used either directly or after wetting. In single addition experiments, a dose of 30% wt. of linalool was dissolved in different amounts of solvent/dispersant (from 62 L, top row, to 500 L, bottom row). When 5 successive encapsulation cycles were performed (5 rep.s), the final amount of linalool corresponded to a theoretical loading of 150%; in order to use the same color-coded scale used for the single addition experiments, EE% and LC% are presented separately for this set of experiments. For this screening, n=3.

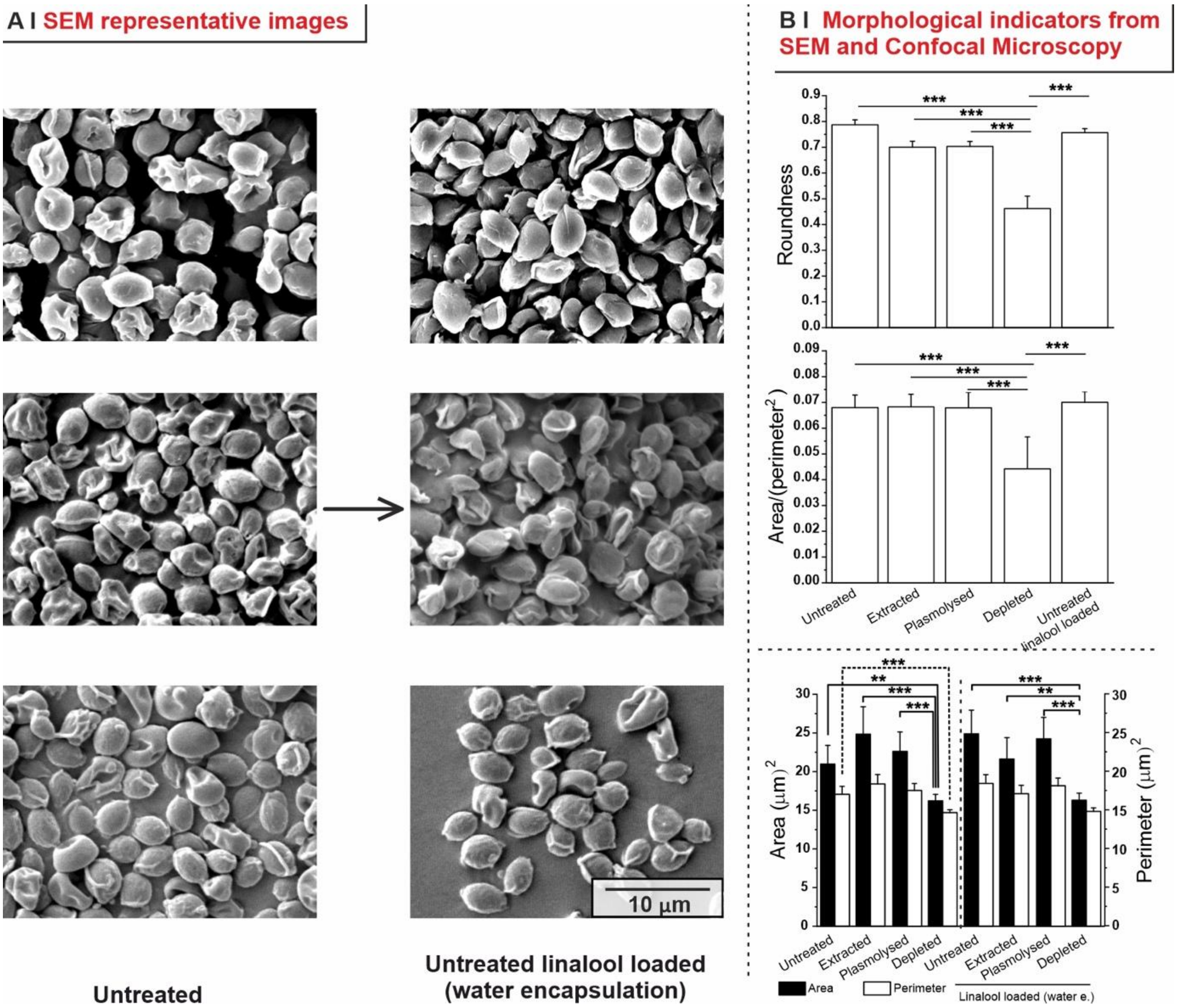

Figure S7. ***A.*** SEM images of yeast cells do not highlight any significant change in the morphological parameters taken into account (area, perimeter, roundness and qualitative cell wall smoothness) for the freeze dried products following encapsulation of hydrophobes (here: linalool). ***B.*** Numerical indicators gathered from SEM (*top and middle graph*) and confocal microscopy (*bottom graph*) images show no significant shape and size difference between cells having undergone the pre-treatments and the linalool-loaded counterparts, with the exception of depleted cells. In SEM images, the roundness (*top graph*; ratio between minor and major axes, equal to 1 for perfect spheres; One-Way ANOVA, $13<n<70$, p=0.001***;) and the ratio area/perimeter^2 (*middle graph*, giving $1/4\pi \approx 0.08$ for perfect spheres) show a somehow less than spherical (oval) geometry for all samples (e.g. for roundness p=0.05 between untreated, extracted or plasmolyzed cells), except depleted cells, which are statistically different (One-Way ANOVA; n=40, p=0.001***) and more flattened. A similar conclusion can be reached using the confocal microscopy data (*bottom graph*): the only statistically relevant differences are against depleted cells, which show considerably smaller areas than any other pre-treated cells either with (right) or without (left) linalool loading (One-Way ANOVA with $15<n<70$, p=0.001***, p=0.01**).

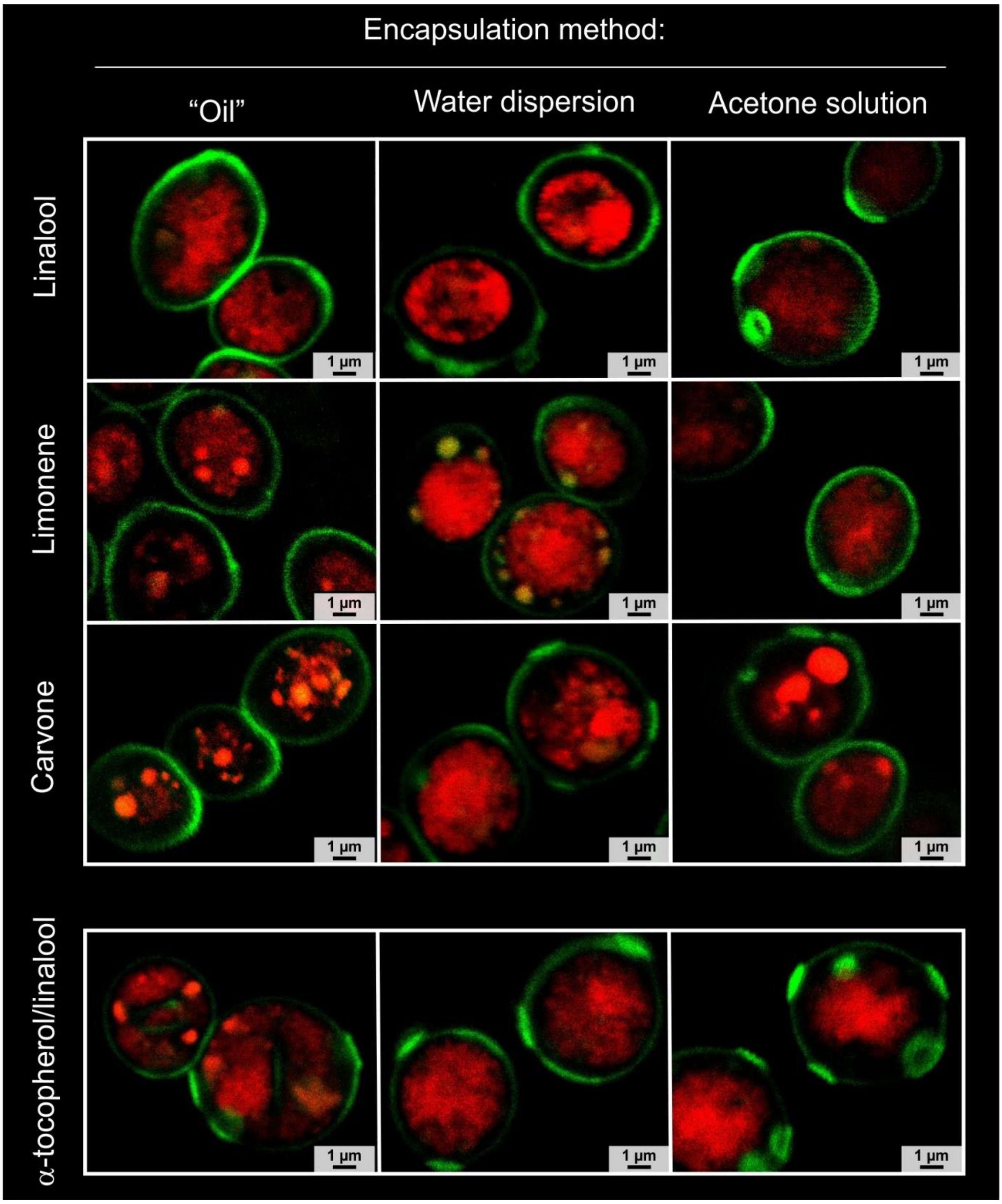

Figure S8. CLSM pictures of untreated yeast cells (stained in red with Nile Red for hydrophobic content and in green with Concanavalin A-Alexa Fluo 488 for cell wall mannans) according to f the hydrophobic terpene and of the encapsulation method. Of note, the fluorescence of hydrophobes encapsulated with the 'oil' method is most often localized in specific domains or punctuated, above all for limonene and dihydrocarvone, while that resulting from the encapsulation in acetone is always dispersed through most of the cytoplasm due to higher contents of analyte.

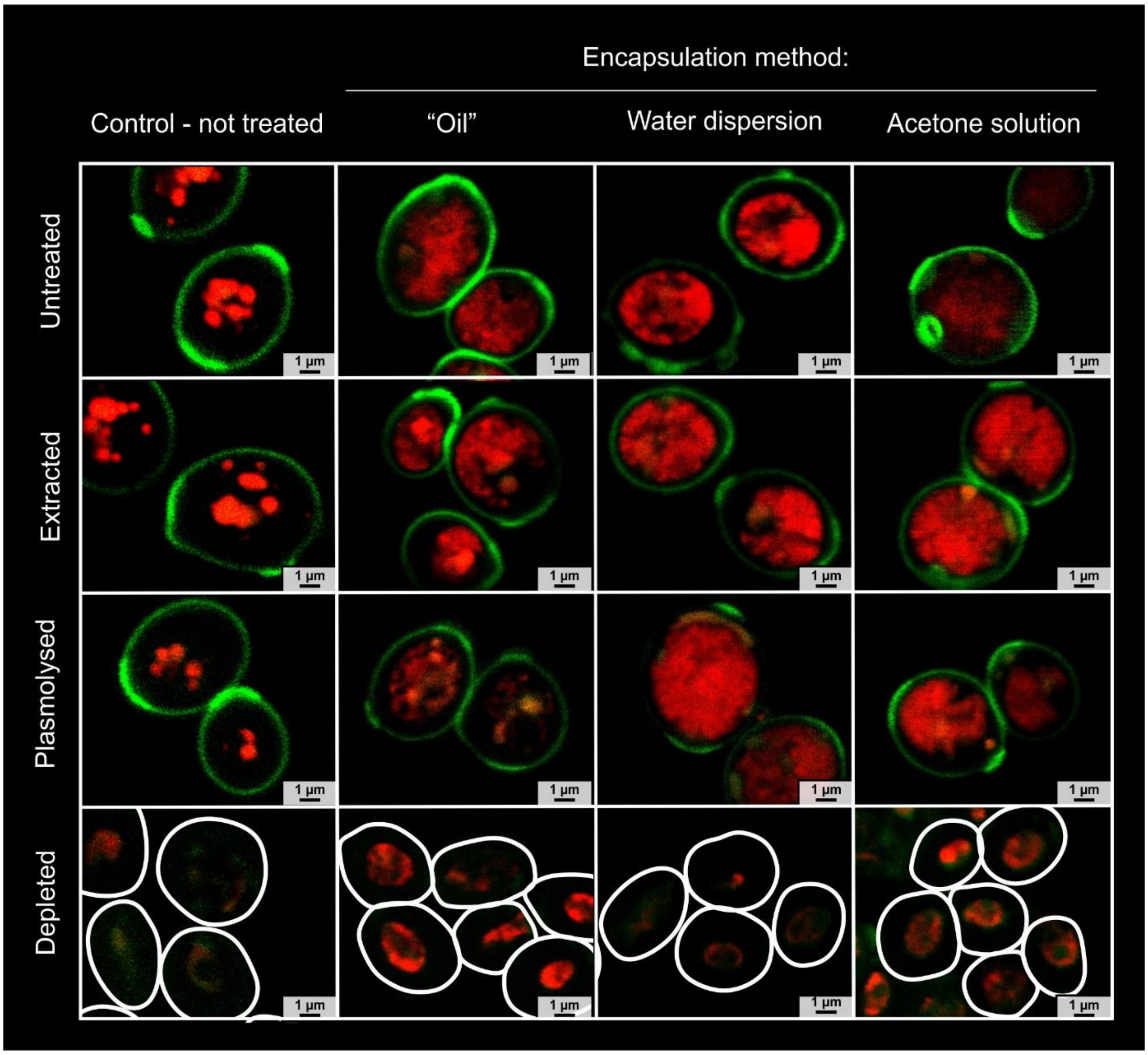

Figure S9. CLSM pictures of pre-treated yeast cells (stained in red with Nile Red for hydrophobic content and in green with Concanavalin A-Alexa Fluo 488 for cell wall mannans) exposed to linalool with the three different encapsulation methods adopted in this work. Of note, Concanavalin A did not stain heavily the walls of depleted cells, because the basic treatment has likely quantitatively cleaved mannose residues. For these cells, bright field images are used to recognize their borders, which are then highlighted in white here.

Table S5. Loading capacity (LC%) data in the encapsulation of single hydrophobes before freeze drying. Standard deviation is expressed for technical replicates, *n*=3.

Process	Untreated			Extracted			Plasmolyzed			Depleted		
	Linal.	Dihyd.	Lim.	Linal.	Dihyd.	Lim.	Linal.	Dihyd.	Lim.	Linal.	Dihyd.	Lim.
'Oil'	24.60 ±0.04	19.95 ±0.09	4.30 ±0.07	15.08 ±0.05	4.82 ±0.06	3.69 ±0.06	18.09 ±0.03	11.4 ±0.2	3.21 ±0.04	4.73 ±0.04	0.97 ±0.09	1.5 ±0.1
Water disp.	15.31 ±0.02	15.37 ±0.04	1.728 ±0.008	18.64 ±0.05	16.70 ±0.09	2.27 ±0.01	9.197 ±0.009	5.554 ±0.006	7.49 ±0.01	2.492 ±0.006	1.201 ±0.001	0.382 ±0.002
Acetone solution	21.9 ±0.1	15.44 ±0.08	11.61 ±0.07	14.63 ±0.05	18.3 ±0.1	10.24 ±0.07	19.14 ±0.04	16.4 ±0.1	6.70 ±0.02	4.42 ±0.01	0.91 ±0.01	1.87 ±0.03

Table S6. Loading capacity (LC%) data in the encapsulation of single hydrophobes after freeze drying, corresponding to data in Figure 6. Standard deviation is expressed for technical replicates, *n*=3.

Process	Untreated			Extracted			Plasmolyzed			Depleted		
	Linal.	Dihyd.	Lim.	Linal.	Dihyd.	Lim.	Linal.	Dihyd.	Lim.	Linal.	Dihyd.	Lim.
'Oil'	24.7 ±0.2	14.63 ±0.09	4.1 ±0.1	12.10 ±0.05	2.44 ±0.06	1.19 ±0.03	14.33 ±0.09	3.15 ±0.07	1.56 ±0.06	0.44 ±0.03	0.98 ±0.06	0.4 ±0.1
Water disp.	14.73 ±0.02	11.03 ±0.06	1.69 ±0.01	17.98 ±0.04	15.40 ±0.05	1.75 ±0.01	9.361 ±0.004	5.338 ±0.001	6.95 ±0.03	0.287 ±0.005	0.396 ±0.003	0.036 ±0.006
Acetone solution	18.8 ±0.1	15.38 ±0.07	10.31 ±0.02	13.98 ±0.08	18.1 ±0.1	11.19 ±0.06	19.2 ±0.1	15.4 ±0.1	5.898 ±0.009	0.21 ±0.03	0.55 ±0.02	1.85 ±0.03

S6. Encapsulation of α-tocopherol/linalool mixture

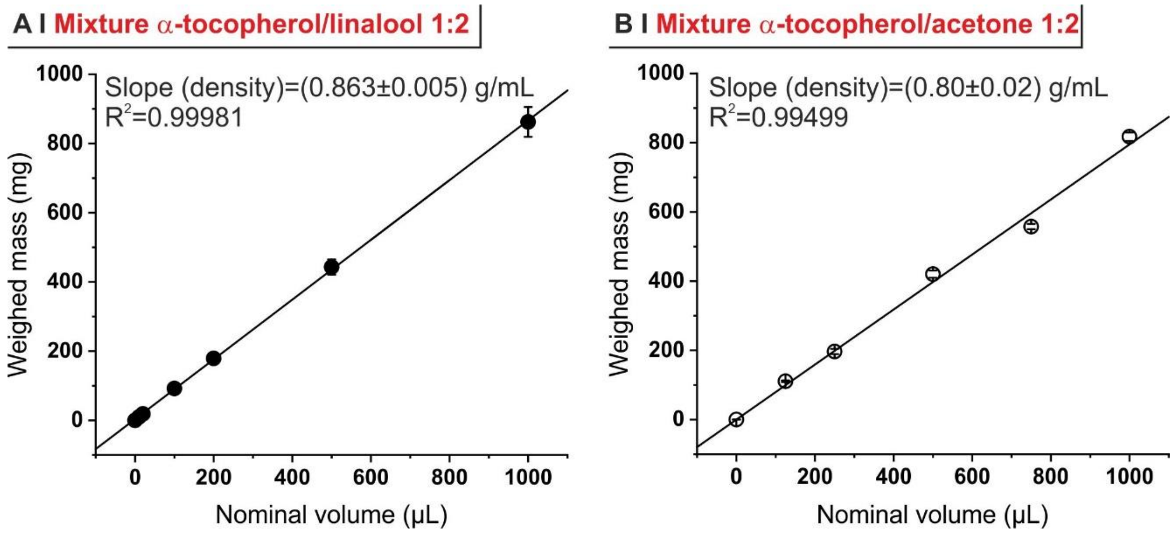

Figure S10. Both graphs represent the weighed mass *vs*. nominal volume (as obtained from pipetting) for **A.** a 1:2 wt. α-tocopherol/linalool solution or **B.** a 1:2 wt. α-tocopherol/acetone solution; the slope of the linear fitting is directly the density of the mixture. n = 5. Densities were used to convert hydrophobe masses to volumes that could be added with a micropipette.

Table S7. Numerical values of input and output variables used for categorical PCA.

Hydrophobe	LogP	Pre-treatment	Nile Red D_{app} (10^{-3} $s^{1/2}$)	Process	Medium diel. const. (ε) [a]	EE (%)	Recovery (%)
Dihydrocarvone	3.1	control	3.09	water	66.7	36.78	71.78
Dihydrocarvone	3.1	plasmolyzed	4.21	water	66.7	17.79	96.10
Dihydrocarvone	3.1	EtOH extracted	4.14	water	66.7	51.34	92.21
Dihydrocarvone	3.1	depleted	6.17	water	66.7	1.32	32.95
Linalool	3	control	3.09	water	66.7	49.11	96.23
Linalool	3	plasmolyzed	4.21	water	66.7	31.20	101.78
Linalool	3	EtOH extracted	4.14	water	66.7	59.92	96.44
Linalool	3	depleted	6.17	water	66.7	0.96	11.50
Limonene	4.4	control	3.09	water	66.7	5.64	97.95
Limonene	4.4	plasmolyzed	4.21	water	66.7	23.18	92.81
Limonene	4.4	EtOH extracted	4.14	water	66.7	5.83	77.25
Limonene	4.4	depleted	6.17	water	66.7	0.12	9.34
Dihydrocarvone	3.1	control	3.09	acetone	19.5	62.83	86.24
Dihydrocarvone	3.1	plasmolyzed	4.21	acetone	19.5	51.27	93.54
Dihydrocarvone	3.1	EtOH extracted	4.14	acetone	19.5	60.36	99.07
Dihydrocarvone	3.1	depleted	6.17	acetone	19.5	1.83	60.73
Linalool	3	control	3.09	acetone	19.5	34.37	88.84
Linalool	3	plasmolyzed	4.21	acetone	19.5	63.92	100.17
Linalool	3	EtOH extracted	4.14	acetone	19.5	46.61	95.60
Linalool	3	depleted	6.17	acetone	19.5	0.71	4.84
Limonene	4.4	control	3.09	acetone	19.5	51.28	99.63
Limonene	4.4	plasmolyzed	4.21	acetone	19.5	19.66	87.98
Limonene	4.4	EtOH extracted	4.14	acetone	19.5	37.29	109.20
Limonene	4.4	depleted	6.17	acetone	19.5	6.16	99.06
Dihydrocarvone	3.1	control	3.09	‘’oil’’	2.5	48.77	73.33
Dihydrocarvone	3.1	plasmolyzed	4.21	‘oil’	2.5	10.51	27.67
Dihydrocarvone	3.1	EtOH extracted	4.14	‘oil’	2.5	8.15	50.67
Dihydrocarvone	3.1	depleted	6.17	‘oil’	2.5	3.25	100.95
Linalool	3	control	3.09	‘oil’	2.5	82.26	100.31
Linalool	3	plasmolyzed	4.21	‘oil’	2.5	47.75	79.20
Linalool	3	EtOH extracted	4.14	‘oil’	2.5	40.34	80.25
Linalool	3	depleted	6.17	‘oil’	2.5	1.46	9.28
Limonene	4.4	control	3.09	‘oil’	2.5	4.28	29.81
Limonene	4.4	plasmolyzed	4.21	‘oil’	2.5	5.21	48.63
Limonene	4.4	EtOH extracted	4.14	‘oil’	2.5	4.18	34.02
Limonene	4.4	depleted	3.09	‘oil’	2.5	1.21	24.61

[a] Water: 66.7 [1]; acetone: 19.5 [2]; terpenes, in average: 2.5 [3].

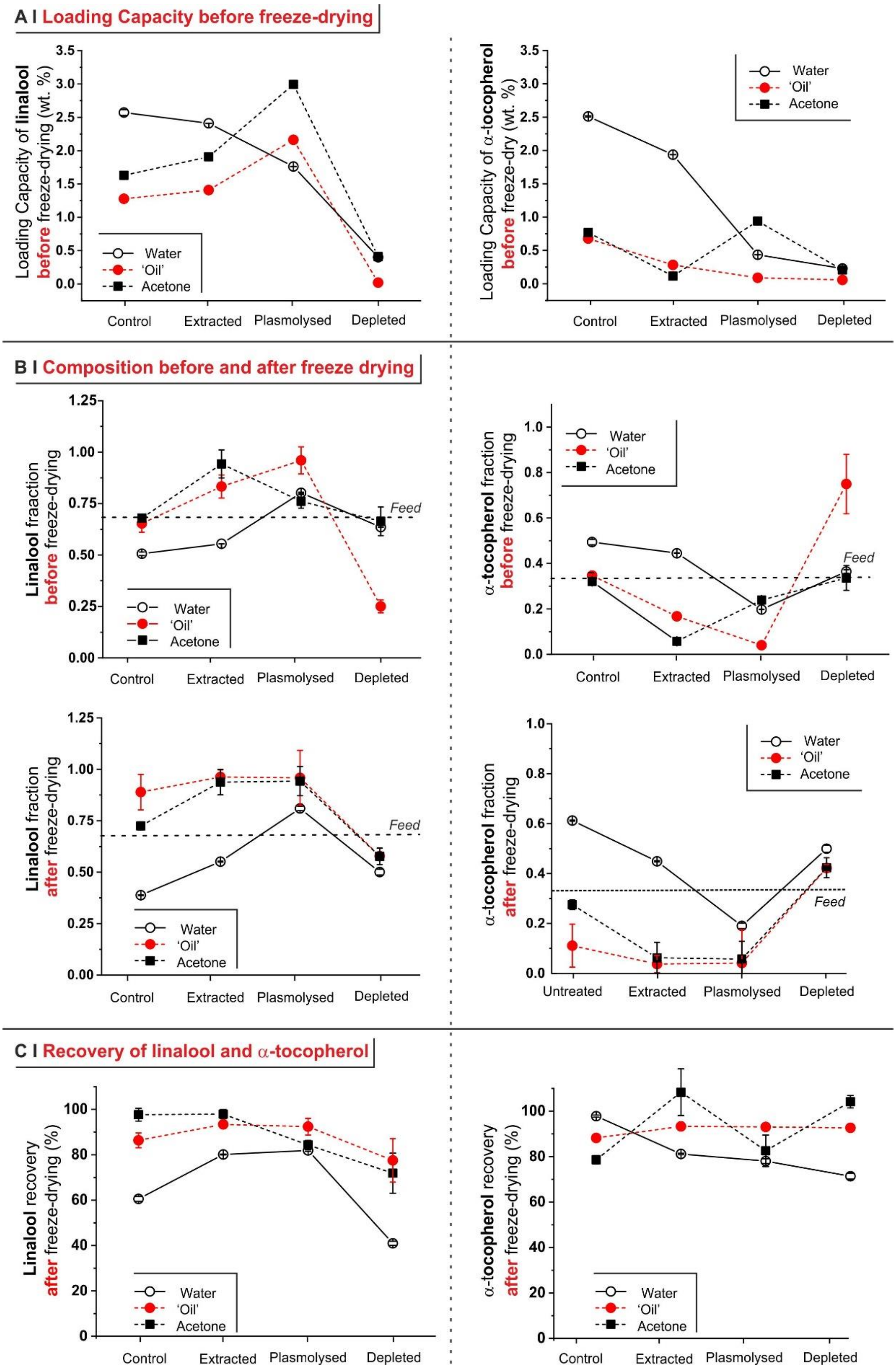

Figure S11. A. Loading capacity of linalool (*left*) and α-tocopherol (*right*) before freeze drying, after their binary encapsulation. **B.** Fraction of linalool (*left*) and α-tocopherol (*right*) in the encapsulated hydrophobe mixture before (*top*) and after (*bottom*) freeze drying. Please note that the encapsulation data of depleted cells are very low, with poor reliability. **C.** Hydrophobes were almost completely retained after freeze drying, except when

encapsulated from water dispersions, which had the highest α-tocopherol loading. These data are also reported in Figure S12.

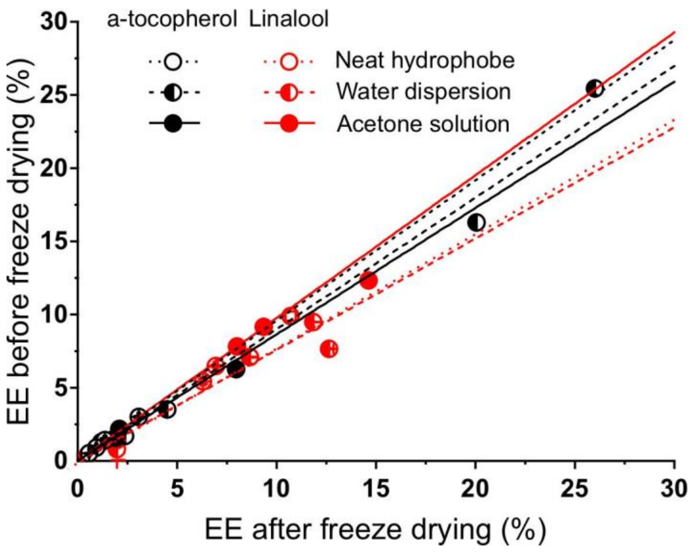

Figure S12. Encapsulation efficiency (EE%) before vs. after freeze drying. This graph shows part of the data of Figure 7B (only binary encapsulation, no depleted cells), reporting them with different symbols for the different processes and showing the corresponding linear fittings.

References

1. Malmberg, C. G.; Maryott, A. A., Dielectric constant of water from 0° to 100° C. *J. Res. Nat. Bur. Stand.* **1956,** 56, 2641–2649
2. Akerlof, G., Dielectric constants of some organic solvent-water mixtures at various temperatures. *Journal of the American Chemical Society* **1932,** 54, (11), 4125-4139 10.1021/ja01350a001.
3. Thomas, G. A.; Hawkins, J. E., Physical and Thermodynamic Properties of Terpenes. IV. The Dielectric Constant, Refractive Index and Density of Some Terpenes1. *Journal of the American Chemical Society* **1954,** 76, (19), 4856-4858 10.1021/ja01648a026.
